# *Escherichia coli* CrfC Protein, a Nucleoid Partition Factor, Localizes to Nucleoid Poles via the Activities of Specific Nucleoid-Associated Proteins

**DOI:** 10.3389/fmicb.2019.00072

**Published:** 2019-02-07

**Authors:** Saki Taniguchi, Kazutoshi Kasho, Shogo Ozaki, Tsutomu Katayama

**Affiliations:** Department of Molecular Biology, Graduate School of Pharmaceutical Sciences, Kyushu University, Fukuoka, Japan

**Keywords:** chromosome partition factor, subcellular localization of protein, nucleoid poles, nucleoid-associated proteins, nucleoid structure, DnaA

## Abstract

The *Escherichia coli* CrfC protein is an important regulator of nucleoid positioning and equipartition. Previously we revealed that CrfC homo-oligomers bind the clamp, a DNA-binding subunit of the DNA polymerase III holoenzyme, promoting colocalization of the sister replication forks, which ensures the nucleoid equipartition. In addition, CrfC localizes at the cell pole-proximal loci via an unknown mechanism. Here, we demonstrate that CrfC localizes to the distinct subnucleoid structures termed nucleoid poles (the cell pole-proximal nucleoid-edges) even in elongated cells as well as in wild-type cells. Systematic analysis of the nucleoid-associated proteins (NAPs) and related proteins revealed that HU, the most abundant NAP, and SlmA, the nucleoid occlusion factor regulating the localization of cell division apparatus, promote the specific localization of CrfC foci. When the replication initiator DnaA was inactivated, SlmA and HU were required for formation of CrfC foci. In contrast, when the replication initiation was inhibited with a specific mutant of the helicase-loader DnaC, CrfC foci were sustained independently of SlmA and HU. H-NS, which forms clusters on AT-rich DNA regions, promotes formation of CrfC foci as well as transcriptional regulation of *crfC*. In addition, MukB, the chromosomal structure mainetanice protein, and SeqA, a hemimethylated nascent DNA region-binding protein, moderately stimulated formation of CrfC foci. However, IHF, a structural homolog of HU, MatP, the replication terminus-binding protein, Dps, a stress-response factor, and FtsZ, an SlmA-interacting factor in cell division apparatus, little or only slightly affected CrfC foci formation and localization. Taken together, these findings suggest a novel and unique mechanism that CrfC localizes to the nucleoid poles in two steps, assembly and recruitment, dependent upon HU, MukB, SeqA, and SlmA, which is stimulated directly or indirectly by H-NS and DnaA. These factors might concordantly affect specific nucleoid substructures. Also, these nucleoid dynamics might be significant in the role for CrfC in chromosome partition.

## Introduction

The bacterial chromosome is organized into a condensed structure called the nucleoid (Wang et al., 2013). The dynamic nature of nucleoids is important for cell growth processes including partitioning of nucleoids. A large number of proteins are involved in the nucleoid dynamics, and many of these proteins localize to the specific subcellular positions (Adachi et al., 2008; Surovtsev and Jacobs-Wagner, 2018). In bacteria, the nucleoid occupies a large percentage of the volume of the cell (Wang et al., 2013). These observations suggest that protein localization dynamics are coupled with the nucleoid dynamics, but the mechanisms underlying this coupling remain elusive.

In *Escherichia coli*, MukB, a SMC (structural maintenance of chromosomes) protein (Nolivos and Sherratt, 2014; Eeftens and Dekker, 2017), plays an important role in nucleoid organization. MukB binds MukEF, forming a ring-like complex that traps DNA strands within the ring (Niki and Yano, 2016). This complex plays essential roles in nucleoid positioning and equipartition (Niki et al., 1991; Hiraga, 2000; Danilova et al., 2007).

The nucleoid-associated proteins (NAPs) of *E. coli*, such as HU (heat unstable protein), H-NS (heat-stable nucleoid-structuring protein), IHF (integration host factor), and Dps (DNA-binding protein from starved cells), bind DNA and contribute to various cellular activities including chromosomal compaction and gene expression (Luijsterburg et al., 2006; Dillon and Dorman, 2010). HU, a highly abundant NAP binds to DNA without sequence specificity, resulting in DNA bending with various angles (Ali Azam et al., 1999; Luijsterburg et al., 2006; Dillon and Dorman, 2010). *E. coli* HU consists of two subunits, HUα and HUβ (encoded by *hupA* and *hupB*, respectively), which form homo- or heterodimers depending on the growth phase (Claret and Rouviere-Yaniv, 1997); in log phase, heterodimers are predominant. HU is distributed throughout the entire nucleoid and plays roles in chromosomal compaction and transcriptional regulation (Wery et al., 2001; Ohniwa et al., 2013; Berger et al., 2016). HU interacts with the replication initiation factor DnaA, stimulating replication initiation at the origin *oriC*, possibly by stabilizing the assembly of DnaA on *oriC* (Chodavarapu et al., 2008a). The Δ*hupA*, Δ*hupB*, and Δ*hupAB* mutations disturb the timing of replication initiation, moderately inhibiting initiation (Bahloul et al., 2001).

IHF, a structural homologue of HU, forms a heterodimer consisting of the IHFα and IHFβ subunits (Luijsterburg et al., 2006; Dillon and Dorman, 2010). Unlike HU, IHF binds to a specific DNA sequence, resulting in sharp DNA bending (Rice et al., 1996). IHF plays important roles in the initiation of DNA replication at *oriC*, DNA recombination at specific sites, and transcription of specific genes (Miller and Friedman, 1980; Arfin et al., 2000; Katayama et al., 2017). IHF is also important for negative regulation of replication: by inactivating a regulator system that downregulates DnaA activity, deletion of IHF causes asynchronous initiation and over-replication of chromosomes (von Freiesleben et al., 2000; Kasho and Katayama, 2013). HU can support initiation at *oriC* in the absence of IHF (Kano and Imamoto, 1990; Hwang and Kornberg, 1992).

H-NS is conserved among Gram-negative bacteria (Dillon and Dorman, 2010). H-NS preferentially binds to AT-rich DNA sequences, constructs multimers, and regulates expression of specific genes, mainly acting as a transcriptional repressor for genes integrated into the genome by horizontal transfer (Dorman, 2004; Lang et al., 2007; Dillon and Dorman, 2010). H-NS multimers are thought to contribute to nucleoid compaction and organization by bridging distant DNA segments (Dame et al., 2006; Japaridze et al., 2017). In the context of nucleoid construction, specific chromosomal regions might be recruited in H-NS multimers (Wang et al., 2011).

Dps, the sequence-nonspecific DNA-binding protein, is an abundant NAP both in stationary phase and under stress conditions, e.g., oxidative, osmotic, acid, or thermal stress (Ali Azam et al., 1999; Calhoun and Kwon, 2011). In addition, Dps may inhibit the DnaA-dependent unwinding of *oriC* by interacting with DnaA (Chodavarapu et al., 2008b); *dps* mutant cells cause a slight enhancement in replication initiation.

The *E. coli* chromosome is organized into several discrete structured subdomains: four macrodomains (Ori, Ter, Left, and Right) and two non-structure regions that rely on arrangement of the long-range chromosomal contacts (Niki et al., 2000; Valens et al., 2004). The Ori macrodomain contains *oriC* and the *maoS* site to which MaoP binds for construction of this macrodomain (Valens et al., 2016). The Ter macrodomain, which contains the replication terminus *terC*, is organized by the MatP protein and its binding sites called *matS*: MatP binds and bridges *matS* sites present in this macrodomain, resulting in the folding of this macrodomain (Mercier et al., 2008; Espéli et al., 2012; Dupaigne et al., 2012). The subcellular positions of these macrodomains are dynamically regulated throughout the cell cycle (Bates and Kleckner, 2005; Youngren et al., 2014).

The structure of the nucleoid is also important for the regulation of cell division. In bacteria, FtsZ is an essential cell division factor that forms a constriction ring (Z-ring) at mid-cell (Haeusser and Margolin, 2016). Assembly of the division machinery on the Z-ring is required for cell division (Haeusser and Margolin, 2016). SlmA (synthetic lethal with a defective Min system) binds to specific DNA sequences called SBSs (SlmA-binding sites) and is localized throughout the nucleoid except within the Ter macrodomain (Cho et al., 2011; Tonthat et al., 2011). SlmA interacts with FtsZ and prevents division-induced chromosomal cutting by inhibiting Z-ring formation over the nucleoid (Bernhardt and de Boer, 2005; Cho et al., 2011).

**FIGURE 1:**
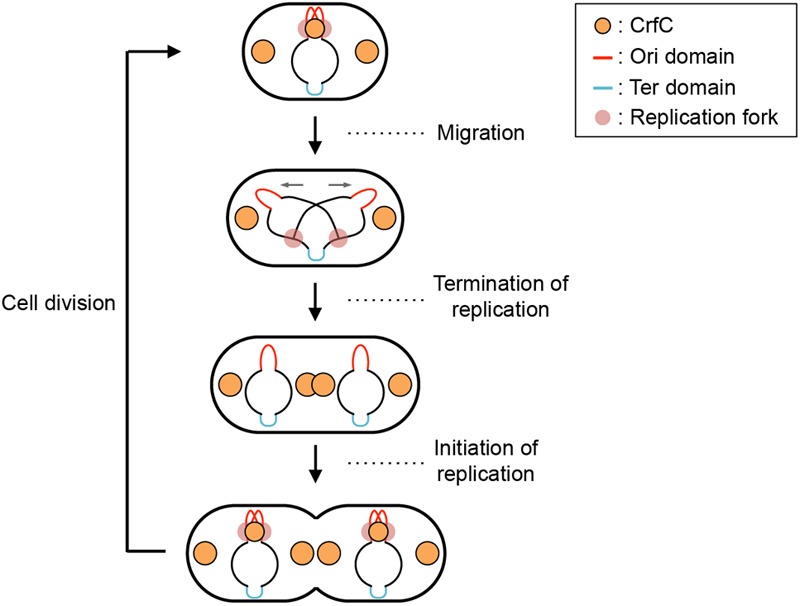
Subcellular localization pattern of CrfC. Schematic illustrations of CrfC when cells are grown at 25°C in M9glu-caa medium. Replication intermediates and subcellular CrfC localization are shown (Ozaki et al., 2013). Sister replication forks are transiently colocalized after the initiation of replication and then CrfC colocalizes with nascent DNA regions at mid-cell. CrfC oligomers bind to the β clamp molecules of the DNA polymerase III holoenzyme, and CrfC molecules localize at cell pole-proximal loci throughout the cell cycle. In addition, prior to cell division, new CrfC foci are produced at mid-cell that will reside at cell pole-proximal loci after cell division (Ozaki et al., 2013).

In *E. coli*, replication of chromosomal DNA is initiated at *oriC* which binds the initiator DnaA protein (Kaguni, 2011; Leonard and Grimwade, 2015; Katayama et al., 2017). DnaA binding promotes unwinding of the *oriC* region, which is followed by loading of DnaB helicase with the aid of the helicase-loader DnaC, resulting in construction of sister replication forks for bidirectional replication. In live cells, the sister replication forks temporally colocalize (Figure 1, top figure) (Sunako et al., 2001; Fossum et al., 2007). The sister nascent DNA regions also transiently colocalize, and after a while, the sister replication forks undergo rapid bidirectional segregation (Figure 1, top to second figures) (Sunako et al., 2001; Bates and Kleckner, 2005; Fossum et al., 2007; Adachi et al., 2008). SeqA (sequestration protein), a hemimethylated DNA-binding protein, is one of the factors supporting colocalization of the sister replication forks (Hiraga, 2000; Fossum et al., 2007). This protein binds to newly replicated DNA regions (Waldminghaus et al., 2012). Also, binding of this protein to *oriC* prevents untimely initiations (Waldminghaus and Skarstad, 2009). Under experimental conditions which we used previously (Ozaki et al., 2013), chromosomal replication is initiated in the segregated sister nucleoids (Figure 1, bottom figure). The chromosomal DNA is synthesized by DNA polymerase (pol) III holoenzyme, which contains the pol III^∗^ subassembly and the β clamp (O’Donnell, 2006). The β clamp is loaded onto the replicating DNA strands to stabilize interaction between pol III^∗^ and DNA strands, and remains on the nascent DNA region after DNA synthesis.

CrfC (colocalization of replication fork DNA by the clamp) protein is a regulator of nucleoid positioning (Ozaki et al., 2013). CrfC has a β clamp–binding motif at the N-terminus (41-QLALP) and a dynamin-like GTPase domain but lacks the typical membrane-binding motif. Like dynamin (Bramkamp, 2012), CrfC forms homomultimers, from dimers to higher-order oligomers. CrfC oligomers bind multiple β clamp molecules (Ozaki et al., 2013). The Δ*crfC* mutant as well as *crfC* Q41A mutant which is defective in β clamp binding produces anucleate cells. In addition, we demonstrated that, in the Δ*crfC* mutant, even the mid-cell positioning of the nucleoid is disturbed in a considerable proportion of growing cells. Also, we showed that cells with a single nucleoid typically contain three CrfC-GFPuv4 foci at the mid-cell and quarter-cell positions (Figure 1, top figure). Intensive analysis of the mid-cell CrfC foci revealed that, immediately after replication initiation, CrfC molecules temporarily colocalize with nascent DNA regions (or the clamp foci) in a manner dependent on the β clamp–binding motif, stabilizing colocalization of the sister replication forks (or the β clamp–bound nascent DNA regions) (Figure 1, top to second figures). In the Δ*crfC* cells, the sister replication forks separate prematurely, soon after replication initiation, leading to defects in equipartition of nucleoids (Ozaki et al., 2013). In addition to the temporal localization at mid-cell, CrfC localizes at the quarter-cell positions throughout the cell cycle (Figure 1). Consistently, in the *crfC* Q41A mutant, formation of CrfC foci is inhibited at the mid-cell, but is sustained at quarter-cell positions, indicating that the quarter-cell CrfC foci are formed independently of the clamp (Ozaki et al., 2013). Those CrfC foci have yet to be investigated in detail.

In this study, we found that the quarter-cell CrfC foci localized near the nucleoid edges, which are the sites nearest the cell poles (hereafter, we refer to these sites as the nucleoid poles). This specific localization could resemble to centrioles in eukaryotic cells in that those are located near both cell-poles sandwiching the chromosomes. The nucleoid-polar localization of CrfC foci was independent of the distance between the nucleoid and cell poles, suggesting a role for specific nucleoid substructures in the CrfC localization. Consistently, HU, SlmA, H-NS, and SeqA played important roles in regulating formation of CrfC foci or the nucleoid-polar localization of CrfC. In addition, DnaA assisted in the roles for HU and SlmA in CrfC foci formation. On the basis of these observations, we hypothesized that a specific nucleoid substructure organized by those DNA-binding proteins is important for the subcellular dynamics of CrfC.

## Materials and Methods

### Bacterial Strains

The *E. coli* strains, plasmids, and primers used in this study are listed in Supplementary Table S1. MECS91 [MG1655 *crfC-venus frt-kan*], MECS68 [MG1655 *mukB-mCherry frt-kan*], and MECS129 [MG1655 *hupA-cfp frt-kan*] were constructed using the λRED recombination system as described previously (Ozaki et al., 2013). Briefly, DNA fragments including *frt*-flanked *kan* and the gene for Venus, mCherry, or CFP were PCR-amplified from template DNA (pTH1017 for Venus, pTH1161 for mCherry, or pTH59 for CFP) and specific primers (SP140 and GFP-b for *crfC-venus*, SP123 and SP124 for *mukB-mCherry*, or SP186 and SP190 for *hupA-cfp*) (Hatano and Niki, 2010; Ozaki et al., 2013). The resultant DNA fragments were electroporated into MG1655 cells bearing pKD46, which expresses λRED proteins (Datsenko and Wanner, 2000). Transformants with correct chromosomal insertions of the desired fragments were purified, yielding strains MECS91, MECS68, and MECS129. Gene loci were transferred by P1 transduction into MG1655 and its derivatives, and the *kan* gene was removed with plasmid pCP20 (Datsenko and Wanner, 2000), yielding MECS91-K [*crfC-venus*], MECS111-K [*crfC-venus, mukB-mCherry*], MECS129-K [*hupA-cfp*], and MECS133-K [*crfC-venus, hupA-cfp*].

The *mCherry-dnaN* gene flanked by *frt-kan*, described previously (Ozaki et al., 2013), was introduced into MECS91-K cells using P1 transduction, and the *kan* gene was removed with pCP20, yielding MECS115-K. Δ*crf*::*frt-kan* was introduced into MECS129-K cells using P1 transduction, and the *kan* gene was removed with pCP20, yielding MECS157. The *ftsZ84* mutation was introduced into MECS133-K cells by P1 transduction using AZ5159 as the donor strain (Kurokawa et al., 1999), yielding MECS150. The *dnaA46* mutation was introduced into MECS133-K and MECS157-K cells by P1 transduction using MIT125 as the donor strain (Noguchi and Katayama, 2016), yielding MECS136 and MECS159, respectively. Δ*mukB*::*cat* was introduced into MECS91-K by P1 transduction using MYU002 as the donor strain (Ozaki et al., 2013), yielding MECS114. Δ*matP*::*frt-kan* from the Keio collection was introduced into MECS91-K and MECS135 cells by P1 transduction, yielding MECS145 and MECS160, respectively. Δ*dps*::*frt-kan* was introduced into MECS133-K and MECS135 by P1 transduction using KMG10 as the donor strain, yielding MECS176 and MECS172, respectively. Δ*ihfA*::*frt-kan* was introduced into MECS133-K and MECS135 by P1 transduction using KMG5 as the donor strain (Kasho et al., 2014), yielding MECS173 and MECS175, respectively. Δ*slmA*::*cat* was introduced into MECS133-K and MECS135 by P1 transduction using MYU008 as the donor strain (Ozaki et al., 2013), yielding MECS177 and MECS178, respectively. Δ*hupB*::*frt-kan* was introduced into MECS133-K, and MECS135 by P1 transduction using KMG8 as the donor strain, yielding MECS179 and MECS180, respectively. Δ*hns*::*frt-kan* was introduced into MECS133-K and MECS135 by P1 transduction using KX181 as the donor strain, yielding MECS171 and MECS174, respectively. *seqA*::Tn*10* was introduced into MECS133-K by P1 transduction using MIT147 as the donor strain, yielding MECS193. *dnaC2* mutation was introduced into MECS133-K, MECS179-K, and MECS177 by P1 transduction using KYA018 or EYK37 as the donor strain (Kasho et al., 2014), yielding MECS195, MECS196 and MECS197, respectively.

### Plasmids and Primers

Plasmids and primers used in this study are listed in Supplementary Tables S2 and S3, respectively.

### Fluorescent Microscopy Analysis in Living Cells

Cells were grown at 25°C in M9 medium supplemented with 0.2% glucose, 0.2% casamino acids, and 5 μg/mL vitamin B1 (M9glu-caa) to an A_660_ of 0.05–0.1. The cells were harvested by brief centrifugation, washed with fresh medium, spotted onto poly-L-Lysine coated slide glass, and observed on a fluorescence microscope (Eclipse 80i; Nikon) equipped with a digital camera system (DP70; Olympus).

For *dnaA46* and *ftsZ84* strains, cells growing at 25°C in M9glu-caa medium were shifted to 42°C and incubated for an additional 2 h. MECS133-K cells were incubated at 25°C in the presence of cephalexin (50 μg/mL) for 2 h. The cells were then prepared and observed as described above.

Phase-contrast or DIC images of cells were merged with fluorescence images using the ImageJ software^1^. Fluorescence intensities in cells were normalized and plotted using the MATLAB-based program Microbe Tracker (Sliusarenko et al., 2011). The subcellular position of the nucleoid pole was identified as the locus where the fluorescence intensity was 50% of the highest signal intensity in the cell.

### RT-qPCR

Cells were grown as described in the previous section. Total RNA was isolated using NucleoSpin RNA (Macherey-Nagel). Aliquots (1 μg) of isolated RNA were treated with DNase I (1 unit; NEB) at 37°C for 10 min, followed by inactivation of DNase I by incubation at 75°C for 10 min. The resultant samples (100 ng) were analyzed by RT-qPCR using 1 μM of specific primers (RT-rpoA-L and RT-rpoA-U for *rpoA*, SP234 and SP235 for *crfC*, or SP256 and SP257 for *slmA*) and One-Step SYBR Green RT-qPCR mixture (Takara). The level of *rpoA* mRNA in each sample was used to normalize the level of *crfC* or *slmA* mRNA. Assays were performed in duplicate.

### Flow Cytometry

Flow cytometry was performed as described previously with minor modifications (Keyamura et al., 2007). Briefly, cells were grown in M9glu-caa medium at 25 or 42°C, followed by incubation in the presence of rifampicin (300 μg/mL) and cephalexin (10 μg/mL) for an additional 4 h, except for *dnaA46* strains, which were incubated at 42°C in the absence of drugs. Cells were collected in cold 70% ethanol; washed; resuspended in cold buffer containing 10 mM Tris-HCl (pH 7.5), 10 mM magnesium sulfate, and 2 μM SYTOX green (Invitrogen); and analyzed on a FACScalibur flow cytometry system (Becton Dickinson).

## Results

### CrfC Foci at Cell-Polar Areas Reside at the Nucleoid Poles

To facilitate investigation of CrfC foci, we constructed a new fluorescently labeled CrfC fusion, CrfC-Venus (Supplementary Figure S1A), as described in “Materials and Methods.” This fusion was designed to emit fluorescence more stably than a previous version, CrfC-GFPuv4 (Ozaki et al., 2013). Flow cytometry analysis revealed that cell cycle regulation was basically intact in cells in which the chromosomal *crfC* gene was replaced with *crfC-venus*, grown in M9 medium containing 0.2% glucose and 0.2% casamino acids (M9glu-caa) (Supplementary Figure S1C). Colocalization of mid-cell CrfC-Venus and clamp-mCherry foci was also sustained, as previously observed for CrfC-GFPuv4 (Supplementary Figure S2) (Ozaki et al., 2013). In this study, we focused on observations of CrfC foci located out of mid-cell.

We speculated that CrfC molecules external to the mid-nucleoid could interact with both poles of the nucleoid, thereby regulating their positions within the cells. Based on this idea, we analyzed the spatial relationship between the CrfC foci and the nucleoid. To analyze nucleoid position, we fluorescently labeled HU protein by replacing the *hupA* gene on the chromosome with *hupA-cfp* (for strain construction, see Supplementary Figure S1B and “Material and Methods”). Flow cytometry analysis confirmed that cell cycle regulation was basically intact in *crfC-venus hupA-cfp* cells grown in M9glu-caa (Supplementary Figure S1C).

**FIGURE 2:**
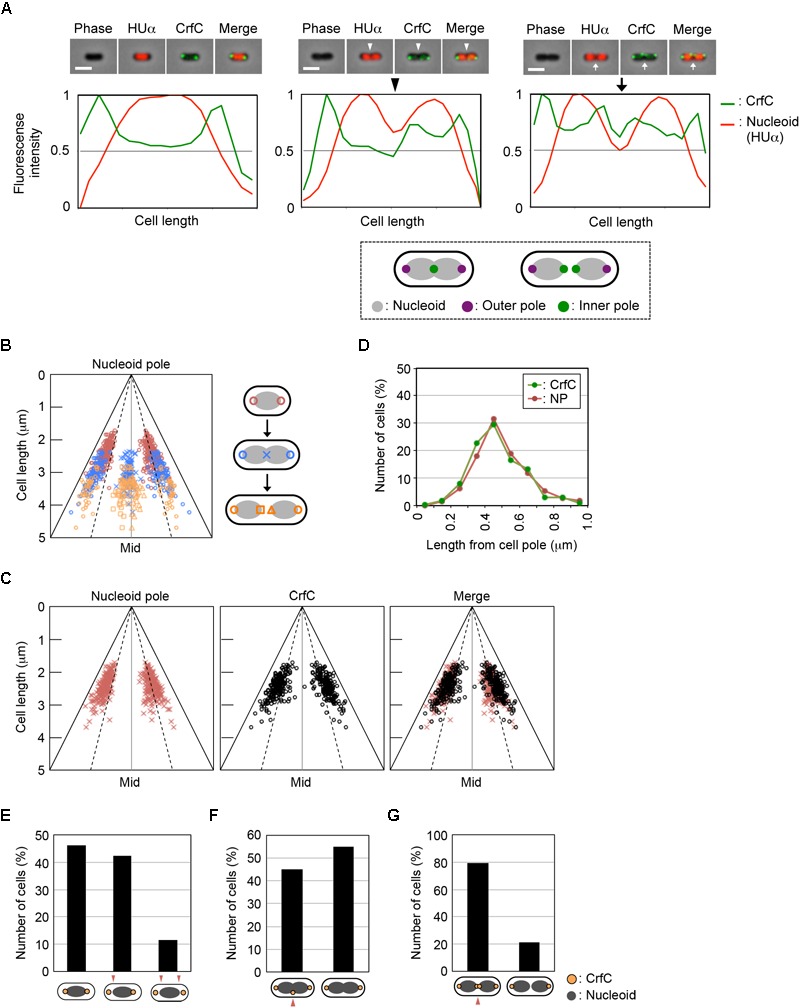
CrfC in the cell-polar area localizes next to the pole of the nucleoid. Fluorescence microscopic analysis of MECS133-K (MG1655 *crfC-venus hupA-cfp*) cells to detect CrfC-Venus and nucleoid foci. Cells were grown exponentially at 25°C in M9glu-caa medium. **(A)** Snapshot imaging of CrfC-Venus and nucleoid (HUα-CFP) in cells. Normalized fluorescence intensities of CrfC (green line) and the nucleoid (red line) along the length of the cell are shown below each cell image. Scale bar is 2 μm. Representative images of cells containing a single nucleoid (left panel), a constricted nucleoid (middle panel), or two nucleoids (right panel) are shown. The positions of the outer nucleoid pole (purple circles) and inner nucleoid pole (green circles) are shown in the lower panel. **(B)** Subcellular positioning of the cell poles in MECS133-K cells. Dotted lines indicate the quarter-cell positions, and black solid lines indicate the positions of the cell poles. Magenta circles indicate nucleoid poles in cells bearing a single nucleoid. Blue circles indicate nucleid poles in cells bearing a constricted nucleoid. Blue cross marks indicate the constriction site of a nucleoid in cells bearing a constricted nucleoid. Orange circles indicate the outer nucleoid poles in cells bearing two nucleoids. Orange squares and triangles indicate the inner nucleoid poles in cells bearing two nucleoids. In total, 326 cells were analyzed. Mid, mid-cell. **(C)** Subcellular positioning of nucleoid poles (left panel) and cell-polar area CrfC (middle panel) in MECS133-K cells containing a single nucleoid. The merged graph is shown in the right panel. In total, 191 cells were analyzed. Mid, mid-cell. **(D)** Distance from the cell pole to the cell-polar area CrfC or the nucleoid pole in the cells shown in **(C)**. Relative CrfC or the nucleoid pole positions were analyzed, and the percentages of cells with the indicated positions are shown. NP, nucleoid pole. **(E)** Histogram of cells with CrfC foci at positions adjacent to or apart from the nucleoid poles in cells analyzed in **(C)**. Proportions (%) of cells with two, one or no CrfC foci adjacent to the nucleoid poles are shown. **(F)** Histogram of the percentage of cells with CrfC foci at the constriction site of the nucleoid. In total, 96 cells bearing a constricted nucleoid were analyzed. **(G)** Histogram of the percentage of cells with CrfC foci at an inter-nucleoid gap. In total, 39 cells bearing two nucleoids were analyzed.

We observed the fluorescence intensities of the double-labeled cells, and quantified the intensity along the long cellular axis (Figure 2A). In single-nucleoid cells with two CrfC foci, the CrfC foci were located at both poles of the nucleoid, typified by the image shown in Figure 2A, left panel. In cells with two partitioned nucleoids, three or four CrfC foci were present: two located at both outer poles of the two nucleoids (Figure 2A, center and right panels), and a single focus or pair of the foci at mid-cell, in the vicinity of the inner poles of the two nucleoids (Figure 2A, center and right panels). These overall features were supported by statistical analysis (Figure 2B,C).

Measurement of the distance between CrfC foci and the nucleoid poles in the single-nucleoid cells revealed that the mean distance was within 0.2 μm in cells with 2–4 μm overall cell length (Figure 2D and Supplementary Figure S3A), and that at least one of the two CrfC foci resided in this range (Figure 2E). There, CrfC foci might have vibration by thermal motion or by temporal structural changes of the nucleoid poles (e.g., via transcription), causing temporal dissociation from the nucleoid poles. Similar analysis using cells with a constricted nucleoid indicated that about 45% of observed cells had CrfC foci near the nucleoid constriction site (Figure 2F). Similar analysis using two-nucleoids cells revealed that a majority of cells had CrfC foci at a site between the sister nucleoids (Figure 2G). Moreover, the overall number of CrfC foci increased as nucleoid segregation progressed (Supplementary Figure S3B). Together, these observations suggest that CrfC foci are newly born during the process of nucleoid migration, and subsequently reside between sister nucleoids, as we previously suggested (Ozaki et al., 2013).

### Subcellular Localizations of CrfC and MukB

It is possible that a subset of the nucleoid-polar CrfC foci might overlap with the bacterial condensin MukB. Previous studies revealed that in cells growing in a minimum medium, MukB preferentially forms two distinct foci at both quarter-cell positions or near the nucleoid poles although the foci numbers and their localization patterns could differ under various growth conditions (Adachi et al., 2008). Therefore, CrfC and MukB molecules could engage in interactions that promote equipartition of the nucleoids.

**FIGURE 3:**
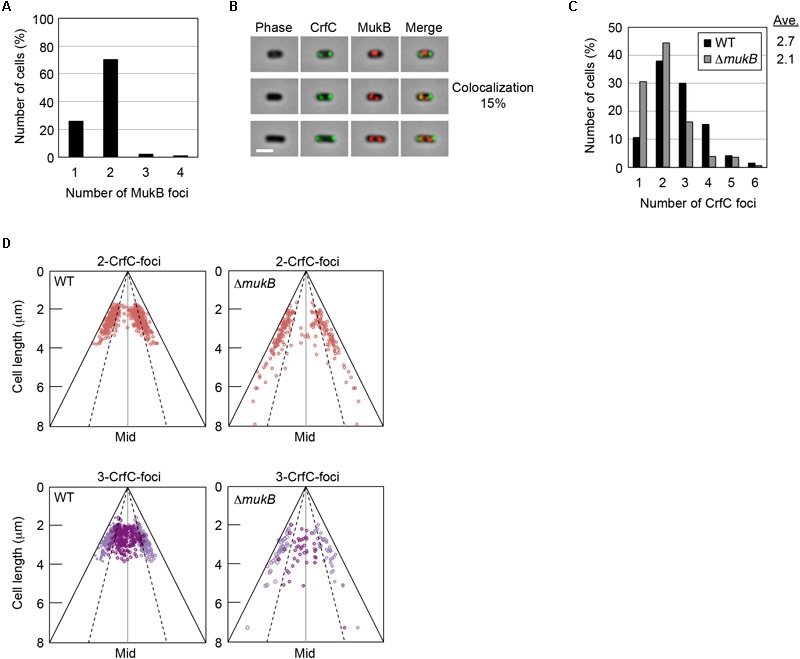
Subcellular localization of CrfC and MukB. **(A)** Percentages of MECS111-K cells containing one to four MukB foci. Cells with more than four foci were not detected. In total, 316 cells were analyzed. **(B)** Representative images of fluorescent foci and the morphology of MECS111-K cells. Cells containing one MukB focus (upper panel), two MukB foci colocalizing with CrfC (middle panel), or two MukB foci not colocalizing with CrfC foci (lower panel) are shown. Scale bar is 2 μm. **(C)** Percentages of MECS91-K (WT; black bars) and MECS144 (Δ*mukB*; gray bars) cells containing one to six CrfC foci. In total, 665 (WT) and 245 (Δ*mukB*) cells were analyzed. Ave; average number of CrfC foci. **(D)** Subcellular positioning of CrfC in MECS91-K (WT) and MECS144 (Δ*mukB*) cells. Cells bearing two CrfC foci (upper panel) or three CrfC foci (lower panel) were analyzed. Mid, mid-cell.

To investigate this possibility, we constructed *crfC-venus* cells in which the chromosomal *mukB* gene was replaced with *mukB-mCherry*. When the cells were grown at 25°C in M9glu-caa, the majority (∼70%) of cells contained two MukB foci at the quarter-cell positions, whereas a minor population contained a single MukB focus at mid-cell (Figure 3A and Supplementary Figure S4). In the cells with two MukB foci, the majority contained two CrfC foci at the quarter-cell positions; however, only 15% of cells exhibited colocalization of one or more MukB and CrfC foci (Figure 3B). Thus, we infer that stable colocalization of MukB and CrfC is unlikely, but we cannot exclude the possibility that a minor population of MukB and CrfC interact at the same positions. The low-frequency colocalization may have occurred by chance due to the dynamic behavior of these foci in live cells. Notably, the CrfC foci in most cells were at positions flanking or outside of the MukB foci (Figure 3B). These observations support the idea that CrfC foci predominantly reside at nucleoid-polar positions, distinct from the MukB positions located inside the nucleoid.

In addition, we quantitatively analyzed the number of CrfC foci and subcellular positions in cells with or without Δ*mukB*. CrfC-Venus cells growing at 25°C in M9glu-caa contained one to six foci per cell, with most cells containing two or three foci (Figure 3C), consistent with data obtained previously using CrfC-GFPuv4 (Ozaki et al., 2013). In terms of these features, *mukB* wild-type cells were similar to Δ*mukB* cells, although cells with single CrfC foci were more abundant, and the average number of CrfC foci was moderately reduced (i.e., 2.1 in Δ*mukB* cells vs. 2.7 in wild-type cells), potentially due to compromised nucleoid dynamics resulting from the Δ*mukB* mutation (Figure 3C) (see “Discussion”). Moreover, the Δ*mukB* cells were slightly elongated, and CrfC was localized at the nucleoid-polar region as in wild-type cells (Figure 3D and Supplementary Figure S1C). Consistently, MukB sustains normal subcellular localization in a Δ*crfC* strain (Supplementary Figure S4). Thus, these results suggest that the subcellular localizations of MukB and CrfC are regulated basically independently.

### Nucleoid-Polar CrfC Location in Elongated *dnaA46* Cells

**FIGURE 4:**
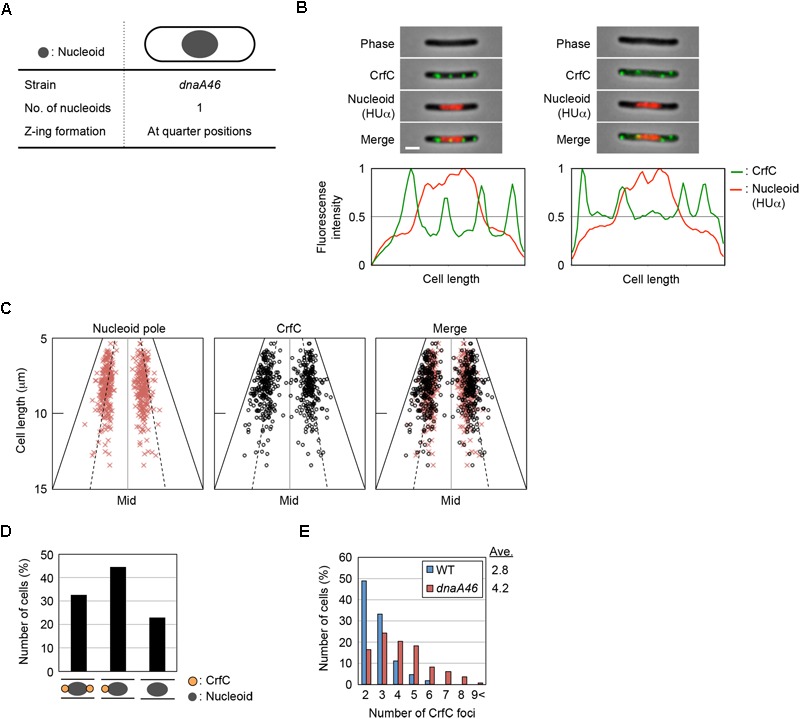
Localization of CrfC and the nucleoid in *dnaA46* filamentous cells. **(A)** Characteristics of *dnaA46* cell incubated at the non-permissive temperature (42°C). When *dnaA46* cells growing at the permissive temperature were transferred to 42°C and then incubated for a few more hours, the majority of cells contained a single nucleoid at mid-cell, and cell division (but not transcription and translation) was inhibited, resulting in elongated cells with an elongated space between the nucleoid poles and cell poles. Gray circle indicates the nucleoid. **(B)** Localization of CrfC foci and the nucleoid in MECS135 (*dnaA46*) cells incubated at 42°C for 2 h after growing exponentially at 25°C. Cells were analyzed by fluorescence microscopy, and the normalized fluorescence intensities of CrfC foci (green line) and the nucleoid (red line) along the length of the cell were plotted. Images of cells with four CrfC foci are shown in the upper panels, and the corresponding fluorescence intensities are shown in the lower panels. Scale bar is 2 μm. **(C)** Subcellular positioning of nucleoid poles and the CrfC foci adjacent to each nucleoid pole in MECS135 (*dnaA46*) cells. Left panel: positions of the nucleoid poles; middle panel: positions of CrfC foci adjacent to each nucleoid pole; right panel: merge of left and middle graphs. The quarter-cell positions are indicated by dotted lines. Mid, mid-cell. **(D)** Percentages of cells with two, one, or no CrfC foci at each nucleoid pole in MECS135 (*dnaA46*) cells. **(E)** Percentages of MECS133-K [wild-type *dnaA* (WT)] or MECS135 (*dnaA46*) cells containing the indicated number of CrfC foci. Numbers of cells analyzed: 326 (WT) and 236 (*dnaA46*). Ave; the average number of CrfC foci.

To more analyze CrfC localization in greater detail, we used *dnaA46* (Ts) cells, which form elongated cells at restrictive high temperatures (Mulder and Woldringh, 1989). We reasoned that analysis of elongated cells should reveal subcellular CrfC localization more clearly than in shorter wild-type cells. When *dnaA46* cells growing at permissive temperature are shifted to a restrictive temperature and incubated for a few hours, they form elongated cells due to inhibition of chromosomal replication initiation concomitant with continued protein synthesis, followed by inhibition of cell division by cells containing a single chromosome (Mulder and Woldringh, 1989; Gullbrand and Nordström, 2000). The resultant elongated cells predominantly contain a nucleoid at mid-cell, and have elongated spaces between this nucleoid and the cell poles (Figure 4A). Although Z-rings involved in cell division are formed at the quarter-cell positions within the elongated spaces in *dnaA46* cells (Gullbrand and Nordström, 2000), constriction itself is inhibited. If nucleoid-polar CrfC depends on the nucleoid rather than the cell poles, then the CrfC foci should remain at the nucleoid poles regardless of the expanded space between the cell poles and the nucleoid.

Cells bearing *dnaA46, crfC-venus*, and *hupA-cfp* were grown at 25°C, and then incubated at 42°C for 2 h. Flow cytometry analysis showed that the resultant cells were elongated 1.8–2.5-fold relative to wild-type cells (Supplementary Figure S1D). Microscopic analysis revealed that the *dnaA46* cells predominantly contained a single nucleoid at mid-cell, and that the nucleoid-polar regions contained CrfC foci (Figure 4B,C and Supplementary Figure S5), suggesting that the nucleoid rather than the cell poles is important in determining the localization of CrfC. The proportion of cells with one or two CrfC foci at the nucleoid-polar regions (∼75%) (Figure 4D) was comparable to that of wild-type cells (∼85%) (Figure 2E): the slight reduction in the *dnaA46* cells could be an indirect consequence of cell elongation and modest oscillation of the foci.

In addition, elongated *dnaA46* cells contained a few additional CrfC foci, generally in the cell-polar regions (Figure 4B,E and Supplementary Figure S5A), suggesting that the cell poles could also affect the localization of the extra CrfC foci. The combination of Δ*crfC* and *dnaA46* produced more anucleate cells than each single mutant; i.e., 2.1% in *dnaA46* Δ*crfC* cells vs. 0.27% in each single mutant cells, suggesting that CrfC is important for the coupling of replication with nucleoid positioning (Supplementary Figures S5B,C). Based on the above results, this synthetic effect would be a consequence of the defect in the CrfC function colocalizing the sister replication forks (Ozaki et al., 2013).

**FIGURE 5:**
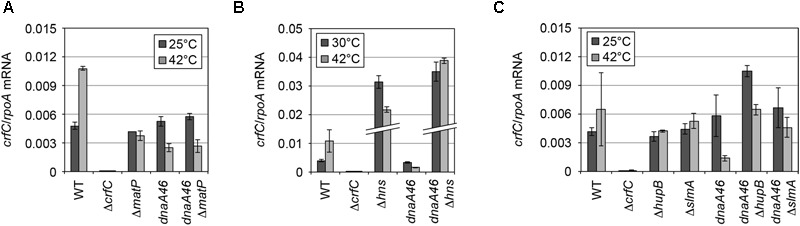
mRNA level of *crfC* in mutant cells. **(A)** Levels of *crfC* mRNA in MECS145 (Δ*matP*) and MECS160 (*dnaA46* Δ*matP*) cells. Cells were grown at 25°C (gray bars) and further incubated at 42°C for 2 h (black bars). Error bars indicate SD (*n* = 2). **(B)** Levels of *crfC* mRNA in MECS171 (Δ*hns*) cells and its derivatives bearing *dnaA46* (MECS174). Cells were grown at 30°C (gray bars) and further incubated at 42°C for 2 h (black bars). Error bars indicate SD (*n* = 2). **(C)** Levels of *crfC* mRNA in Δ*hupB* (MECS179) or Δ*slmA* (MECS177) cells and their derivatives bearing *dnaA46* (MECS180 and MECS178, respectively). Cells were grown at 25°C (gray bars) and further incubated at 42°C for 2 h (black bars). Error bars indicate SD (*n* = 2).

The DnaA protein regulates the transcription of several genes (Messer and Weigel, 1997). At 25°C, the mRNA levels of *crfC* were comparable between wild-type and *dnaA46* cells (Figure 5A). At 42°C, the *crfC* mRNA level was moderately elevated in wild-type cells, but not in *dnaA46* cells. Thus, the slight increase in the number of CrfC foci in *dnaA46* cells could not be explained by upregulation of *crfC* transcription. Also, DnaA could be a transcriptional stimulator of *crfC*. Consistently, the *crfC* promoter region contains DnaA-binding consensus (DnaA box) sequences (Supplementary Figure S6).

### Nucleoid-Polar CrfC Location in Elongated *ftsZ84* Cells

**FIGURE 6:**
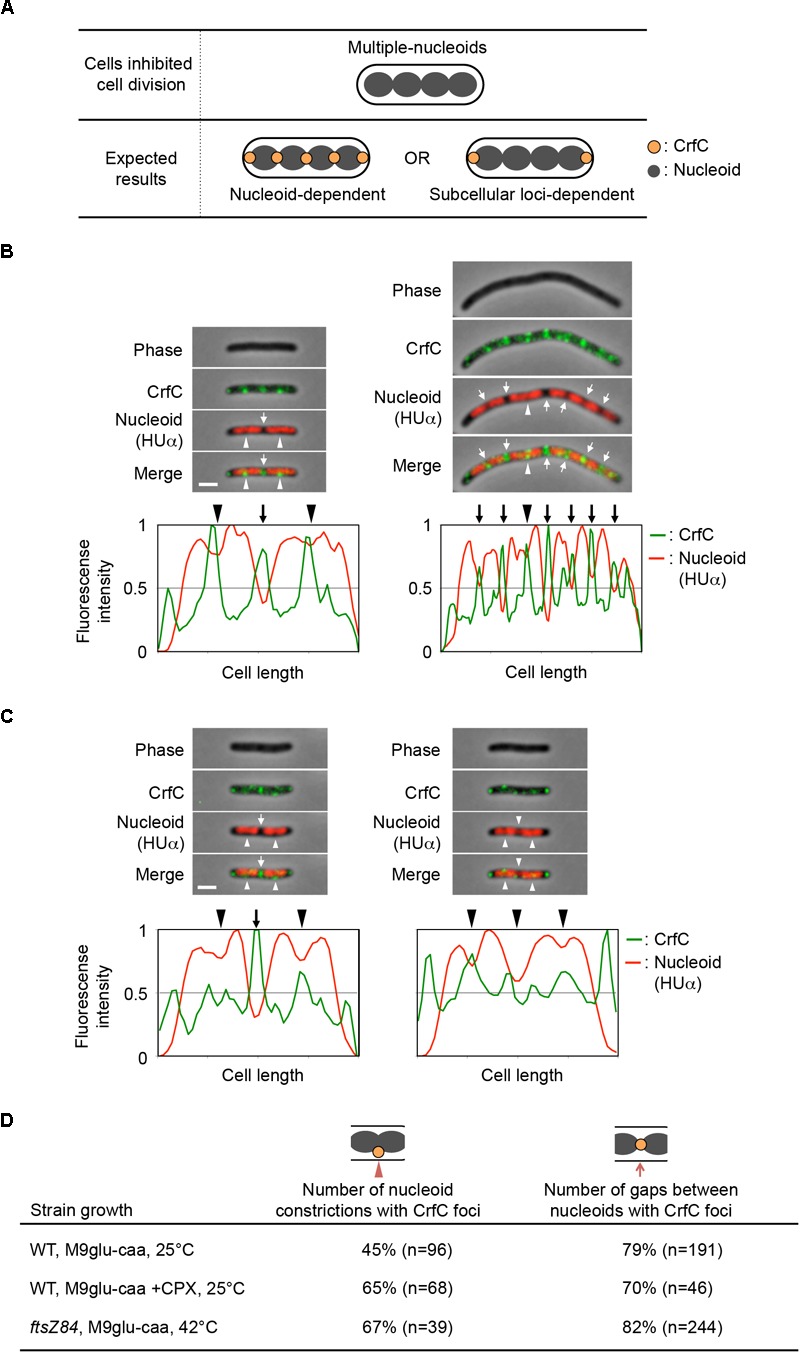
Localization of CrfC and nucleoids in filamentous cells containing multiple nucleoids. **(A)** Characteristics of *ftsZ84* and cephalexin-treated wild-type cells after the inhibition of cell division. The cells were elongated, and replicated nucleoids were partitioned in each cell (upper panel). **(B–C)** Localization of CrfC-Venus and nucleoids in MECS150 (*ftsZ84*) cells **(B)** and cephalexin-treated MECS133-K (wild-type *ftsZ*) cells (C). The *ftsZ84* cells were incubated at 42°C for 2 h and the wild-type *ftsZ* cells were incubated at 25°C for 2 h in the presence of cephalexin. The arrowheads indicate the nucleoid constrictions, and the arrows indicate the inter-nucleoid gaps. Scale bar is 2 μm. Normalized fluorescence intensities of CrfC (green line) and nucleoid (red line) along the length of the cell are shown below each image. **(D)** Percentages of cells with CrfC foci at nucleoid constrictions or at inter-nucleoid gaps in MECS133-K (WT; wild-type), MECS150 (*ftsZ84*), and cephalexin-treated MECS133-K cells. Wild-type data from Figure 2 are shown for comparison. n; number of nucleoid constrictions or inter-nucleoid gaps analyzed. CPX, cephalexin.

As the localization of some cellular proteins is determined by that of the FtsZ rings (Addinall and Lutkenhaus, 1996; Ogino et al., 2004), CrfC localization also could be affected by FtsZ. We used *ftsZ84* (Ts) cells, which form elongated cells at restrictive high temperatures (Addinall et al., 1997); incubation of *ftsZ84* cells at 42°C rapidly breaks the Z-ring, inhibiting cell division. Because chromosomal replication and partition are sustained in the mutant cells, further incubation at 42°C produces elongated cells containing partitioned multiple nucleoids (Figure 6A). In addition, to produce elongated cells by another means, we used cephalexin to inhibit FtsI (penicillin-binding protein 3), which is crucial for cell division. Cephalexin treatment resulted in elongated cells containing multiple nucleoids (Figure 6A), but in this case Z-ring formation is sustained (Pogliano et al., 1997).

Flow cytometry analysis revealed that, when growing *ftsZ84* cells were incubated at 42°C for 2 h, the cells contained 4, 8, or 16 chromosomes each, whereas when growing wild-type cells were incubated with cephalexin at 25°C for 2 h, they contained four or eight chromosomes each (Supplementary Figure S1E). Microscopic analysis showed that the elongated *ftsZ84* cells contained multiple partitioned nucleoids, and that CrfC foci were generally localized at the nucleoid poles (82% of nucleoid poles distant from the cell pole) or at the constriction sites of the separating nucleoids (67% of constricted nucleoids) (Figure 6B,D). These foci were formed even in regions distant from the cell poles. Similar results were observed for the cephalexin-treated cells: CrfC foci were generally localized at the nucleoid poles (70% of nucleoid poles distant from the cell pole) or at the constriction sites of the separating nucleoids (65% of constricted nucleoids) (Figure 6C,D). In addition, *ftsZ84* cells incubated at restrictive high temperatures does not form Z-rings (Addinall et al., 1997), supporting the idea that localization of the Z-ring is not required for regulation of CrfC localization.

### Nucleoid-Polar CrfC Foci Are Stable in Cells Lacking MatP

Next, we considered the possibility that nucleoid structure (e.g., specific folding, constitution and dynamic changes of those in subdomains) could be an important determinant of CrfC localization. The Ter macrodomain, one of the four macrodomains of the *E. coli* chromosome described above, resides in the vicinity of one pole of the nucleoid, except at the time of nucleoid splitting (Bates and Kleckner, 2005; Adachi et al., 2008). Construction of the specific structure of the Ter macrodomain requires the MatP protein and its binding site *matS*. Most of the *matS* sites are concentrated in the Ter macrodomain. Hence, MatP could be related to the nucleoid-polar localization of CrfC.

**FIGURE 7:**
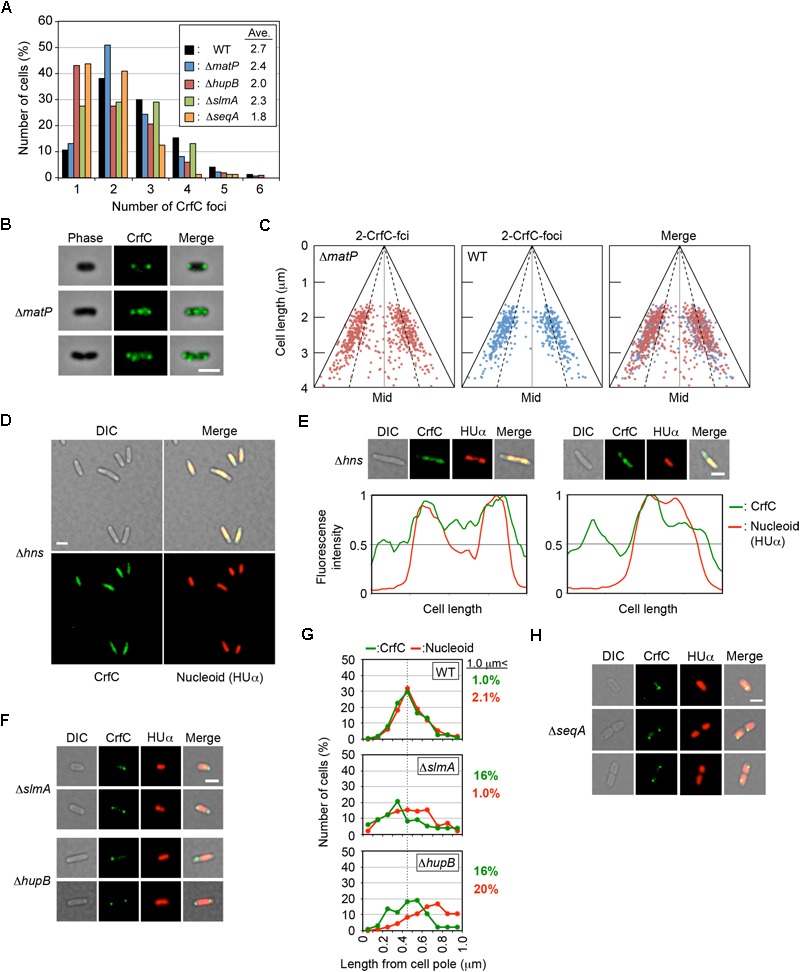
Effect of deletion of nucleoid-associated protein on CrfC localization. **(A)** Percentages of MECS133-K (WT; wild-type control), MECS145 (Δ*matP*), MECS179 (Δ*hupB*), MECS177 (Δ*slmA*), and MECS193 (Δ*seqA*) cells containing the indicated number of CrfC foci. In total, 665 (WT), 663 (Δ*matP*), 102 (Δ*hupB*), 76 (Δ*slmA*), and 71 (Δ*seqA*) cells were analyzed. **(B)** Representative images of fluorescent foci and the morphology of MECS145 (Δ*matP*) cells. Cells containing two or four CrfC foci are shown. Scale bar is 2 μm. **(C)** Subcellular positioning of CrfC in the cell-polar are in MECS91-K (wild-type *matP*) and MECS145 (Δ*matP*) cells. Mid, mid-cell. **(D)** Images of fluorescent foci and the morphology of MECS171 (Δ*hns*) cells incubated at 30°C. Scale bar is 2 μm. **(E)** Representative images of MECS171 (Δ*hns*) cells incubated at 30°C. Scale bar is 2 μm. Normalized fluorescence intensities of CrfC (green line) and nucleoid (red line) along the length of the cell are shown below each image. **(F)** Representative images of fluorescent foci and the morphologies of MECS177 (Δ*slmA*) and MECS179 (Δ*hupB*) cells. Scale bar is 2 μm. **(G)** Distance from the cell pole to the pole-area CrfC or the nucleoid pole in MECS133-K (WT), MECS177 (Δ*slmA*), and MECS179 (Δ*hupB*) cells. NP, nucleoid pole. Broken line: peak position from the graph of wild-type cells. **(H)** Representative images of fluorescent foci and the morphologies of MECS193 (Δ*seqA*) cells. Scale bar is 2 μm.

To analyze the possible role for MatP in CrfC localization, we analyzed Δ*matP* cells incubated at 25°C. The subcellular localization of CrfC and the number of foci were essentially intact in Δ*matP* cells (Figure 7A–C). Only a small increase in the proportion of two-foci cells, and a reduction in the proportion of four-foci cells, were detected in the mutants (Figure 7A), potentially due to a change in nucleoid structure. At 25°C, *crfC* mRNA levels were comparable in Δ*matP* and wild-type cells (Figure 5A). Thus, the involvement of MatP in CrfC localization is unlikely.

### Specific Roles for H-NS

Next, we expanded our mutant analysis to NAPs such as HU, IHF, H-NS, and Dps in addition to SlmA and SeqA. HU is a major protein of NAPs, IHF is a HU homolog with binding sequence specificity, H-NS forms clusters by preferential binding to AT-rich sequences, Dps contributes to DNA compaction, and SlmA sequence-specifically binds to DNA inhibiting Z-ring formation. First, we analyzed single mutants bearing a deletion of one of these proteins. All mutants were grown at 25°C, with the exception of Δ*hns* cells, which are cold-sensitive at 25°C and had to be grown at 30°C.

Notably, in Δ*hns* cells, CrfC molecules were distributed and predominantly colocalized with nucleoids: only a minor population (∼10%) had additional CrfC molecules outside of nucleoids (Figure 7D,E). In addition, the number of chromosomes was reduced in Δ*hns* cells (Supplementary Figure S1F), consistent with previous reports that chromosome replication is moderately inhibited in this mutant (Kaidow et al., 1995; Helgesen et al., 2016).

Because deletion of a nucleoid-associating factor might change the transcriptional activity of the *crfC* gene, we quantified the levels of *crfC* mRNA in each mutant (Figure 5). As for H-NS, repression of *crfC* transcription and binding to the *crfC* promoter region are reported (Oshima et al., 2006; Shimada et al., 2011) (Supplementary Figure S6). Consistently, at 30°C, the *crfC* mRNA level was markedly increased in Δ*hns* cells (Figure 5B). Together, H-NS is crucial for the control of cellular CrfC levels.

When CrfC was overexpressed in cells with pBR322 bearing *crfC*, CrfC molecules were distributed throughout the cell without bias to the nucleoid region, indicating importance of *crfC* expression level for specific localization of CrfC (Supplementary Figures S7A,B). Distribution of CrfC molecules in Δ*hns* cells could be partly explained by increase in the *crfC* expression level. In addition, the predominant localization of CrfC to nucleoids in Δ*hns* cells might be an indirect consequence by nucleoid structural changes and expression of other genes (see also below).

### Specific Roles for SlmA and HU

In contrast to Δ*hns* cells, CrfC formed foci in Δ*slmA* or Δ*hupB* mutant cells (Figure 7F), although the number of CrfC foci per cell was moderately reduced in Δ*hupB* cells (i.e., 2.0 in Δ*hupB* cells and 2.7 in wild-type cells) (Figure 7A). Specifically, the proportion of cells with one CrfC focus was three-fold higher in the mutant than in the wild-type strain (Figure 7A), suggesting that HUβ is important for regulation of CrfC foci formation. In addition, it should be noted that compared with wild-type cells, cells bearing Δ*slmA* or Δ*hupB* contained polar CrfC in a broader region proximal to the cell pole (Figure 7G). Location of the nucleoid pole was slightly broadened in cells bearing Δ*slmA* or Δ*hupB*. At 25°C, the *crfC* mRNA levels in Δ*slmA* or Δ*hupB* cells were similar to those in the wild-type cells (Figure 5C), suggesting that SlmA and HU play important roles in determining CrfC localization in a manner distinct from H-NS. By contrast, CrfC localization in Δ*ihfA* and Δ*dps* cells resembled that in wild-type cells (Supplementary Figure S8).

### Specific Roles for SeqA

SeqA binds to the hemimethylated DNA region which temporally emerges after passage of the replisomes (Waldminghaus and Skarstad, 2009). This protein plays multiple roles in repressing untimely initiations at *oriC* and supporting colocalization of nascent DNA regions (Fossum et al., 2007). In Δ*seqA* cells, CrfC formed foci, but the average number of CrfC foci was reduced; i.e., 1.8 in Δ*seqA* cells vs. 2.7 in wild-type cells (Figure 7A,H). This could be caused partly by destabilization of cololalization of the sister replication forks, inhibiting CrfC foci formation there. Also, CrfC foci formation at the nucleoid poles might be indirectly or directly inhibited by disturbed processes in construction of the nucleoid substructures (see “Discussion”).

### Specific Roles for MatP in *dnaA46* Cells

**FIGURE 8:**
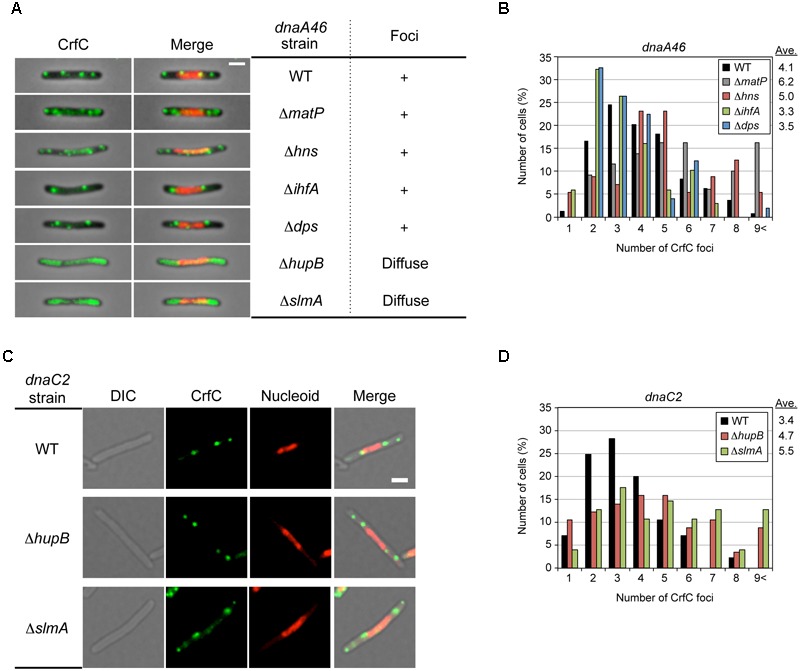
Effect of *dnaA46* double mutation on CrfC localization. **(A)** Subcellular localization of CrfC in MECS135 (*dnaA46*) cells (WT) and its derivatives bearing Δ*matP* (MECS160), Δ*hns* (MECS174), Δ*ihfA* (MECS175), Δ*dps* (MECS172), Δ*hupB* (MECS180), or Δ*slmA* (MECS178). Cells were incubated at 42°C for 2 h. Left panels show representative images of fluorescent foci and the morphologies of *dnaA46* strains. Scale bar is 2 μm. Right panels show the subcellular localization patterns of CrfC in *dnaA46* strains. In certain strains, discrete CrfC foci were constructed (+). In other strains, discrete CrfC foci were not observed, and fluorescence was uniform throughout the cell (Diffuse). **(B)** Percentages of cells of MECS135 (*dnaA46*) (WT) and its derivatives bearing Δ*matP* (MECS160), Δ*dps* (MECS172), Δ*hns* (MECS174), or Δ*ihfA* (MECS175) containing the indicated numbers of CrfC foci. Ave; average number of CrfC foci. In total, 241 (WT), 129(Δ*matP*), 49 (Δ*dps*), 56 (Δ*hns*), and 68 (Δ*ihfA*) cells were analyzed. **(C)** Representative images of fluorescent foci and the morphology of MECS195 (*dnaC2*), MECS196 (*dnaC2* Δ*hupB*) and MECS197 (*dnaC2* Δ*slmA*) filamentous cells. Scale bar is 2 μm. **(D)** Percentages of cells of MECS195 (*dnaC2*) (WT), and its derivatives bearing Δ*hupB* (MECS196), or Δ*slmA* (MECS197) containing the indicated numbers of CrfC foci. Ave; average number of CrfC foci. In total, 85 (WT), 57 (Δ*hupB*), and 102 (Δ*slmA*) cells were analyzed.

To further investigate the role of NAPs for the nucleoid-polar localization of CrfC, we analyzed *dnaA46* cells bearing the mutations of NAPs. The cells were incubated at 42°C, as described for the experiments shown in Figure 4. The resultant cells were elongated (Supplementary Figures S1D,G and S9). In Δ*matP dnaA46* double-mutant cells, the average number of CrfC foci was somewhat higher than in *dnaA46* cells; i.e., 6.2 in Δ*matP dnaA46* cells vs. 2.4 in Δ*matP* cells or 4.1 in *dnaA46* cells (Figure 7A and 8), and those foci were predominantly distributed in spaces between the nucleoid and cell poles. The proportions of cells with one or two CrfC foci at the nucleoid-polar regions were comparable in the Δ*matP dnaA46* mutant (∼75%) and in *dnaA46* cells (∼80%), suggesting that Δ*matP* did not affect regulation for keeping the nucleoid-polar CrfC. At 42°C, the *crfC* mRNA levels were comparable in *dnaA46* Δ*matP* and *dnaA46* cells (Figure 5A). In wild-type cells, *crfC* transcription was increased ∼2-fold at 42°C vs. 25°C, and this change was dependent upon both DnaA and MatP (Figure 5A). These results suggest that regulation of the number of CrfC foci is disturbed by a synthetic effect of combining *dnaA46* and Δ*matP* mutations. The absence of MatP could indirectly aggravate defects of nucleoid substructures caused by *dnaA46* mutation in promoting CrfC foci formation.

### Specific Roles for SlmA, HU and H-NS in *dnaA46* Cells

Similarly, we analyzed *dnaA46* mutant cells bearing the Δ*hupB*, Δ*hns*, Δ*ihfA*, Δ*slmA*, or Δ*dps* mutation. The mutants were grown at 25°C or 30°C, and then shifted to 42°C and incubated for an additional 2 h. The resultant cells were elongated, like *dnaA46* cells, except that the elongation of the *dnaA46* Δ*slmA* double mutant was relatively moderate (Supplementary Figures S1D and S9); consistent with this, Δ*slmA* stimulates cell division in *dnaA* mutant cells (Bernhardt and de Boer, 2005).

Notably, microscopic analysis revealed that, in *dnaA46* Δ*hupB* and *dnaA46* Δ*slmA* cells, CrfC did not form foci; instead, the majority of CrfC was diffused throughout the cellular space, excluding the nucleoid, although only a minor fraction was present even within the nucleoid space (Figure 8A). The *crfC* mRNA levels in *dnaA46* Δ*hupB* and *dnaA46* Δ*slmA* cells at 42°C were comparable to those in wild-type cells (Figure 5C). Moreover, deletion of the *hupB* gene in *dnaA46* cells did not decrease the mRNA levels of *slmA* (Supplementary Figure S10), suggesting that deletion of *hupB* in *dnaA46* cells disturbed CrfC foci formation independently of *slmA* expression. These results further suggest important roles for HU and SlmA in regulation of CrfC foci formation and localization during dynamic structural changes in nucleoids.

*dnaA46* Δ*hns* double-mutant cells contained several CrfC foci colocalized with the nucleoid, but fewer foci in the cell-polar regions (Figure 8A and Supplementary Figure S9), despite the fact that the *crfC* mRNA level was markedly increased even at 42°C (Figure 5B). Given that the Δ*hns* cells contained CrfC molecules distributed throughout the nucleoid, and also had elevated levels of *crfC* mRNA (Figure 5B and 7D,E), these results suggest that introduction of *dnaA46* to Δ*hns* cells rescued a process involved in CrfC foci formation independent of the *crfC* expression level (see “Discussion”). *dnaA46* cells bearing Δ*ihfA* or Δ*dps* exhibited basically normal localization of CrfC foci (Figure 8A,B). Together, our data supported the idea that subcellular dynamics of CrfC relies on multiple factors including DnaA and chromosomal substructures facilitated by distinct NAPs.

In addition, to investigate specificity to DnaA, we similarly analyzed mutant strains bearing *dnaC2* (Ts), which inhibits replication initiation (but not DNA synthesis by the replisomes) and produces elongated cells at restrictive high temperatures as with the *dnaA46* mutant strains (Gullbrand and Nordström, 2000). At 42°C, the localization pattern of CrfC foci in *dnaC2* single-mutant cells was basically similar with that in *dnaA46* single-mutant cells (Figure 8A,C and Supplementary Figure S11). However, unlike the *dnaA46* derivatives, *dnaC2* Δ*hupB* and *dnaC2* Δ*slmA* cells sustained CrfC foci formation (Figure 8C,D and Supplementary Figure S11). This suggests a specific role for DnaA in CrfC foci formation in the absence of *hupB* or *slmA*. *dnaC2* cells had the slightly less number of CrfC foci than *dnaA46* cells and the number in *dnaC2* cells slightly increased by introduction of Δ*hupB* or Δ*slmA* (Figure 8B,D), which could be consequences of indirect complicated effects of DnaA (see “Discussion”).

## Discussion

In this study, we demonstrated that the *E. coli* chromosomal partitioning regulator CrfC localizes at nucleoid-polar regions throughout the cell cycle (Figure 2). Nucleoid exclusion is one of the mechanisms underlying cell-polar localization of diffusible molecules (Reyes-Lamothe et al., 2014; Neeli-Venkata et al., 2016). However, CrfC formed foci at the nucleoid poles even when the size of the nucleoid-free space was increased (Figure 4 and 6). This suggests the idea that CrfC localization to nucleoid-polar regions is independent of nucleoid exclusion mechanisms and even the distance between the nucleoid pole and the cell pole.

**FIGURE 9:**
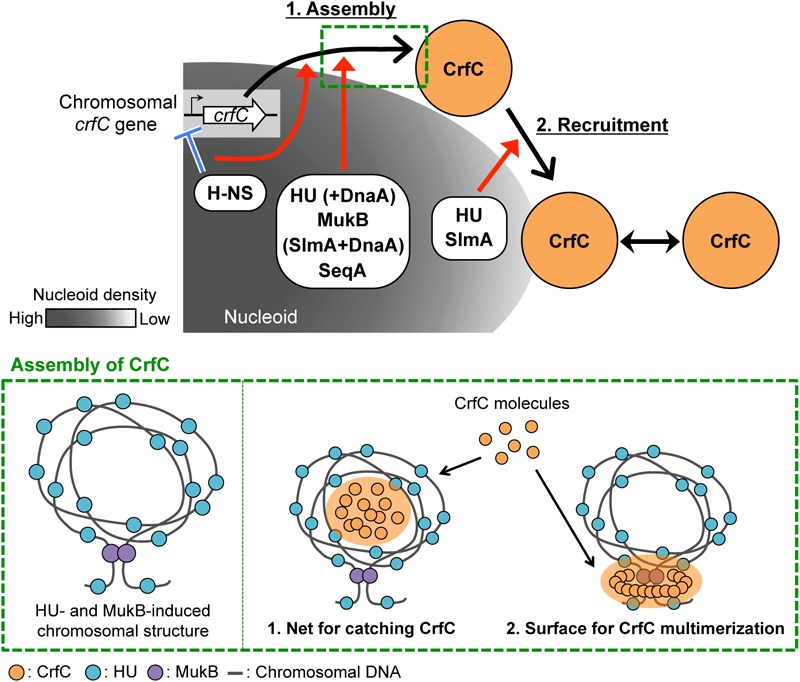
Model of subcellular localization of CrfC. Model of CrfC localization to positions adjacent to the nucleoid pole. We hypothesize that CrfC localization is controlled in a two-step process: (1) assembly and (2) recruitment. H-NS promotes efficient assembly of CrfC by spatiotemporally controlling the CrfC expression and organizing the nucleoid structure. HU and MukB provides the specific nucleoid structure that influences assembly of CrfC by concentrating CrfC molecules or stimulating CrfC multimerization. The lower panel shows possible mechanisms for stimulation of CrfC multimerization. A specific DNA folding is constructed depending on HU and MukB (*left*) (Lioy et al., 2018). CrfC molecules might be concentrated in the DNA folding or might be stimulated in multimerization by interaction with the surface of the DNA folding (*right*) (see the text). DnaA stimulates CrfC assembly either directly or indirectly in the absence of HU or SlmA. Also, SeqA stimulates CrfC assembly. DnaA and SeqA might stimulate functional substructures of the nucleoid. DnaA can interact with HU (Chodavarapu et al., 2008a). CrfC assembly then interacts with the nucleoid pole-specific structure, which is organized by HU and SlmA, directing CrfC localization at the nucleoid poles. There, CrfC foci might have vibrating motion, causing temporal dissociation from the nucleoid pole. These specific nucleoid organization factors cooperatively promote the proper subcellular dynamics of CrfC. Low nucleoid density in the nucleoid-polar region also could be involved.

Consistently, we showed that several NAPs are important for CrfC localization (Table 1, Figure 9). In particular, H-NS represses *crfC* gene transcription and changes the formation of CrfC foci (Table 1, Figure 5B, 7D,E and 8). Notably, HU and SlmA support CrfC localization to the nucleoid-polar regions without significantly affecting *crfC* mRNA levels. In the deletion mutants of HUβ or SlmA, DnaA is required for the formation of CrfC foci (Table 1, Figure 7A,F,G and 8). In addition, MukB stimulates CrfC foci formation but not localization (Table 1, Figure 3C). By contrast, CrfC foci formation and localization at nucleoid-polar regions were supported even in cells lacking MatP, IHFα, or Dps. These results indicate that CrfC localization to nucleoid-polar regions is dependent on a specific nucleoid structure organized by specific NAPs and the related proteins. For example, difference in nucleoid density, distribution of highly-folded chromosomal DNA (Kuhlman and Cox, 2012; Le Gall et al., 2016), might be related (Figure 9). In *E. coli*, nucleoid density is higher in the central region of the nucleoid and lower in the periphery, which is known to be related for localization of specific proteins (Kuhlman and Cox, 2012; Le Gall et al., 2016). Deletion of specific DNA-binding proteins might cause significant changes in density at nucleoid poles, resulting in defects of CrfC localization.

**Table 1:** Summary of results.

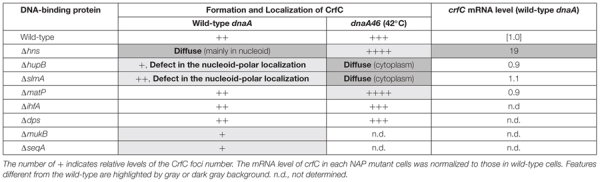

### Specific Roles for NAPs and Related Proteins in CrfC Localization

HU and MukB stimulate the formation of CrfC foci (Table 1, Figure 3C and 7A). Lioy et al. (2018) reported that HU and MukB act cooperatively to promote long-distance (>800 kb) folding of *E. coli* chromosome outside of Ter macrodomain: deletion of HU or MukB decreases long-range chromosomal contacts and increases short-range (∼280 kb) contacts. The HU- and MukB-induced sub-chromosomal structure might affect the assembly of CrfC in the nucleoid periphery (Figure 9). We consider two possibilities: (i) CrfC could be trapped in the “mesh” of a highly-folded chromosome, resulting in the local high concentration of CrfC molecules, and (ii) CrfC multimerization could be stimulated by interaction with DNA folds including HU and MukB, as CrfC is a dynamin homolog and can form multimers (Ozaki et al., 2013) (Figure 9).

Of the two, HU is also important for the nucleoid-polar localization of CrfC foci (Table1, Figure 3D and 7G). The subcellular distribution of HU differs from that of MukB: HU is widely distributed throughout the entire nucleoid (Wery et al., 2001), whereas MukB forms clusters at specific sites even within the nucleoid (Danilova et al., 2007). HU localized at nucleoid-polar regions might stabilize CrfC localization at the nucleoid poles via a direct or indirect interaction (also see below) (Figure 9).

The nucleoid occlusion protein SlmA was also required for the nucleoid-polar localization of CrfC (Table 1, Figure 8G). Like HU, SlmA colocalizes throughout the entire nucleoid, except in the Ter macrodomain (Cho et al., 2011; Tonthat et al., 2011). SlmA binding distorts DNA, allowing cooperative binding of proteins and activation of transcription initiation of specific genes (Tonthat et al., 2013; Klancher et al., 2017). These observations suggest that SlmA organizes a specific nucleoid substructure by inducing the conformational changes in DNA and/or specific DNA–protein complexes. At the nucleoid poles, SlmA and HU might cooperate to promote formation of a nucleoid-polar specific higher-order substructure, which directs CrfC foci localization (Figure 9). We reported previously that double mutation of *crfC* and *slmA* causes a synthetic defect in nucleoid positioning, indicating a genetic interaction between CrfC and SlmA (Ozaki et al., 2013), which might underlie SlmA-CrfC coorganized functions. At 42°C, *dnaA46* Δ*crfC* double-mutant cells were elongated as the *dnaA46* single-mutant cells, whereas *dnaA46* Δ*slmA* double-mutant cells exhibited relatively moderate cell elongation (Supplementary Figure S1D). This is consistent with the idea that SlmA acts as a division inhibitor independently of CrfC. Consistently, our previous study shows that cell division and replication initiation of the chromosome are fundamentally intact in the Δ*crfC* cells (Ozaki et al., 2013).

In *E. coli*, chromosomal replication is an important determinant of chromosome organization (Cagliero et al., 2013). *dnaA46* cells and *dnaC2* cells exhibited basically similar localization pattern of CrfC at 42°C (Figure 8). However, in the absence of *hupB* or *slmA, dnaA46* mutation, but not *dnaC2* mutation, severely inhibited CrfC foci formation (Figure 8), suggesting a specific role for DnaA. DnaA itself might have direct or indirect roles in CrfC localization. As ∼300 DnaA binding sites (DnaA boxes) are suggested to be distributed throughout the genome (Hansen et al., 2007), DnaA binding to those sites might affect construction of specific substructures of the nucleoid, assisting in CrfC foci formation. Also, direct interaction between HU and DnaA, which is reported previously (Chodavarapu et al., 2008a), might contribute to CrfC localization in wild-type or Δ*slmA* cells. Alternatively, as DnaA acts also as a transcriptional regulator of certain genes (Hansen et al., 2007), DnaA-dependent transcription might indirectly stimulate construction of specific nucleoid substructures. The synthetic defects in formation of nucleoid substructures inhibit functional interaction with CrfC resulting in inhibition of CrfC foci formation and diffusion throughout cells (Table 1; Figure 9). Slight differences in the CrfC foci numbers between *dnaC2* cells and *dnaA46* cells could be caused by indirect effects, which remains to be elucidated (Figure 8).

Δ*hns* cells overexpressed *crfC* mRNA and contained diffused CrfC colocalized with nucleoids (Table 1, Figure 5B and 7D,E). The requirement for H-NS in CrfC foci formation could be partly explained by the idea that CrfC must be expressed at an appropriate level, dependent on H-NS activity, to properly assemble. As shown in cells bearing pBR322-*crfC* (Supplementary Figure S7), *crfC* overexpression would be the primary case of CrfC diffusion. Factors (including specific substructures of nucleoids) stimulating CrfC foci formation might be limited in the number, which inhibits functionally interact with excessive CrfC molecules, resulting in total diffusion of CrfC (also see below). In addition to negative regulation by H-NS, expression of *crfC* is suggested to be positively regulated by transcription of the sigma factor FliA (σ^F^), of which binding sites are present in the *crfC* promoter region (Zhao et al., 2007) (Supplementary Figure S6). Furthermore, the present analysis suggests that DnaA and MatP also stimulate *crfC* transcription at 42°C (Figure 5). As the *crfC* promoter region contained several DnaA-binding consensus sequences (but not *matS* sites) (Supplementary Figure S6), DnaA could stimulate the *crfC* transcription directly, or indirectly, by inhibiting H-NS binding to this region. In Δ*matP* cells, defects in structures of nucleoids could indirectly impede stimulation of the *crfC* transcription at 42°C.

In Δ*hns* cells, even extra molecules of CrfC stay with nucleoids, which might be supported by interaction with altered substructures of nucleoid. Altered nucleoid structures as well as expression levels of specific genes in the absence of H-NS might increase affinity of substructures of nucleoids for CrfC, preventing diffusion of CrfC to the cytosol. H-NS could downregulate interaction between CrfC and nucleoid substructures by nucleoid organization. In Δ*hns dnaA46* cells at 42°C, further changes in nucleoid structure could activate alternative pathways for formation and nucleoid-polar localization of CrfC foci. Similar changes in *dnaA46* cells could stimulate CrfC foci formation also in the absence of MatP. Taken together, H-NS is inferred to stimulate formation of CrfC foci (Figure 9) and alternative pathways independent of H-NS would be induced to stimulate CrfC foci formation by the defect of DNA replication or DnaA. These further imply the presence of dynamic interplay between CrfC and nucleoid substructures.

### Subcellular Dynamics and Possible Function of Nucleoid-Polar CrfC

Although further characterizations remain, this study revealed that subcellular CrfC localization requires specific factors involved in regulating nucleoid substructures. Based on our results, we hypothesize that CrfC localization is controlled in a two-step process: (1) assembly and (2) recruitment to the nucleoid poles (Figure 9). In the first step, CrfC is assembled on the nucleoid periphery. H-NS acts as a transcriptional repressor of *crfC*, promoting this step. Also, H-NS might spatiotemporally regulate CrfC dynamics as a nucleoid organizer. HU and MukB assist in this step, which might be based on a cooperatively function of the two in providing the specific nucleoid substructure as described above, stimulating CrfC assembly (Figure 9). Moreover, in the absence of HU or SlmA, DnaA functions assist in CrfC foci formation. DnaA might affect the nucleoid substructures directly or indirectly. Conversely, in the absence of H-NS and MatP, DnaA downregulates CrfC foci formation (Table 1). Also, SeqA stimulates CrfC assembly, which might depend also on affects to the nucleoid substructures, as SeqA binds to nascent DNA regions emerged during chromosome replication (Waldminghaus et al., 2012). In addition, stabilization of colocalization of the sister replication forks by SeqA may contribute to CrfC foci formation at the forks. As such, the CrfC assembly step is assisted in by dynamic interplay between CrfC and nucleoid organization. In the second step, CrfC is recruited to the nucleoid poles, stabilizing its localization. CrfC must recognize a nucleoid pole–specific structure induced by HU and SlmA. Direct interaction of CrfC with HU and SlmA could promote CrfC recruitment to the nucleoid poles (Figure 9). Also, CrfC foci might vibrate in the proximity of the nucleoid poles, causing temporal dissociation (Figure 2). To reveal the detailed mechanism underlying CrfC localization, we are searching for the functional region of CrfC that is required for localization to the nucleoid poles.

As no other nucleoid partition factors are reported to show the localization similar to CrfC, CrfC at the nucleoid poles could play novel and unique roles in nucleoid regulation. For example, nucleoid-polar CrfC foci could contribute to chromosome migration as physical marks; i.e., those could indicate the migration orientation of the newly replicated nucleoids like eukaryotic centrioles during chromosome equipartition. Whereas CrfC present at the replication fork regulates the initial steps of nucleoid equipartition (Ozaki et al., 2013), nucleoid-polar CrfC could indicate the destination for migration of the future sister nucleoids. The other possibility is that CrfC is kept on standby for specific cellular reactions occurring at the nucleoid poles. In *E. coli*, chromosomal DNA damage promotes formation of single-stranded DNA at the cell-polar area (Kohiyama et al., 2013), which is similar to the position of CrfC. CrfC at the nucleoid poles might be involved in reactions necessary for chromosome stability. Further studies are required to elucidate the molecular mechanisms underlying the localization and function of nucleoid-polar CrfC.

## Author Contributions

All authors conceived the experiments and analyzed the data. ST performed the experiments. ST, SO, and TK wrote the manuscript.

## Conflict of Interest Statement

The authors declare that the research was conducted in the absence of any commercial or financial relationships that could be construed as a potential conflict of interest.

## Footnotes

1 https://imagej.nih.gov/ij/
