# Supplementary material for: Escherichia coli CrfC Protein, a Nucleoid Partition Factor, Localizes to Nucleoid Poles via the Activities of Specific Nucleoid-Associated Proteins
Source: Front Microbiol. 2019 Feb 7;10:72. doi: 10.3389/fmicb.2019.00072 (PMC6374313; doi:10.3389/fmicb.2019.00072)
Supplement: Supplementary file 1 [file Data_Sheet_1.pdf]

frontiers

Supplementary Material

Escherichia coli CrfC protein, a nucleoid partition factor, localizes to nucleoid poles via the activities of specific nucleoid-associated proteins

Saki Taniguchi*, Kazutoshi Kasho, Shogo Ozaki, Tsutomu Katayama

*** Correspondence:** Tsutomu Katayama: katayama@phar.kyushu-u.ac.jp.

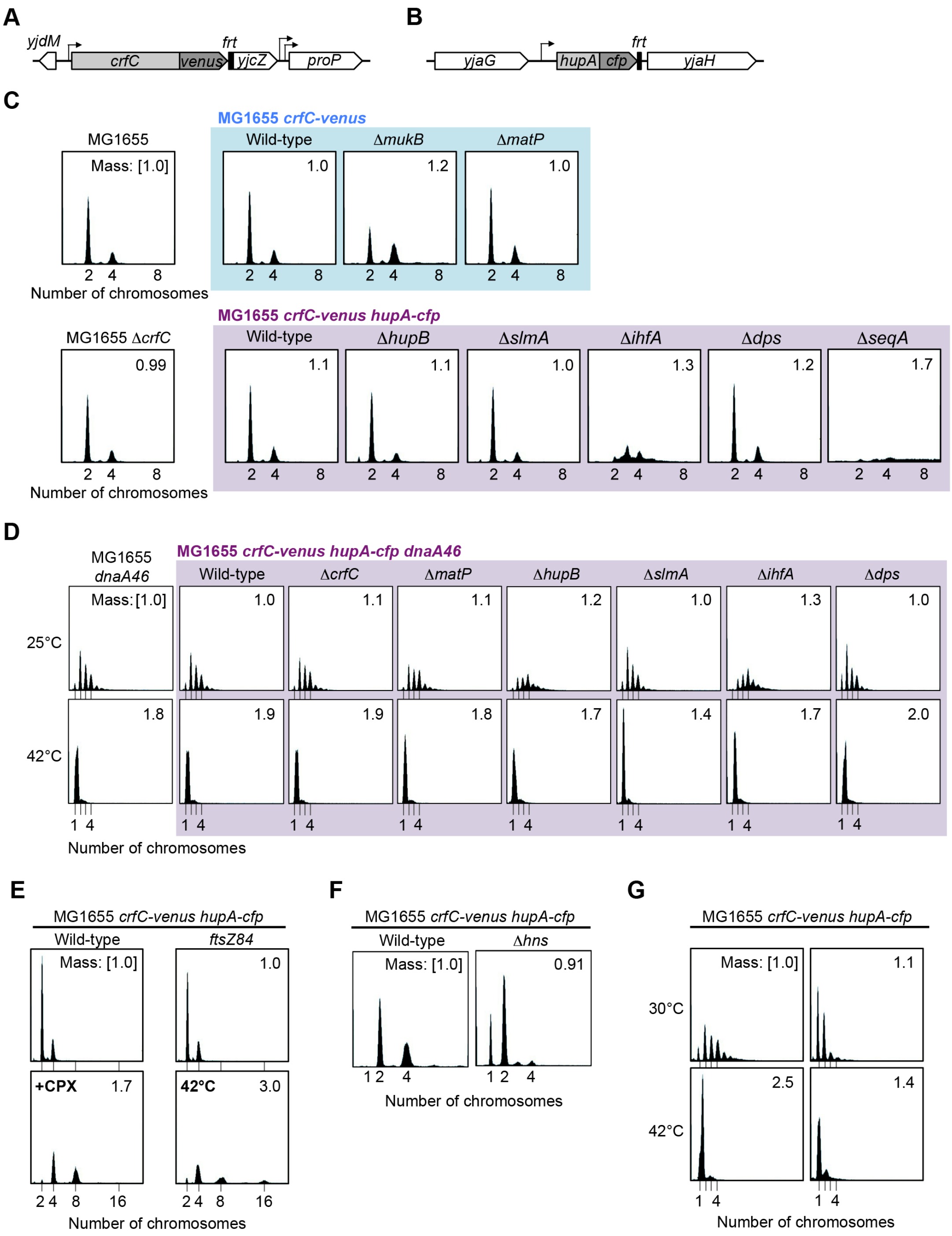

Supplementary Figure 1. Flow cytometry analysis of constructed mutants. (A-B) Illustrations of the chromosomal *crfC* region (A) and *hupA* region (B). (C-G) Flow cytometric analysis of MG1655-derivative strains grown at 25, 30 or 42°C in M9glu-caa medium. Cells were incubated for an additional 4 hr at the same temperatures in the presence of rifampicin and cephalexin, followed by analysis on a FACS Calibur instrument as described previously (Keyamura et al., 2007). (C) MECS91-K, expressing Venus-fused CrfC, and its derivatives. (D) *dnaA46* double mutants other than MECS174 (*dnaA46* Δ*hns*). (E) MECS133-K treated with cephalexin and MECS150 (MECS133-K *ftsZ84*). (F) MECS171 (Δ*hns*) grown at 30°C. (G) MECS174 (*dnaA46* Δ*hns*).

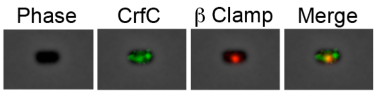

1-clamp focus cells (A)	Colocalized cells (B)	B/A (%)
23	12	52

Supplementary Figure 2. Colocalization of CrfC and β clamp at mid-cell. Colocalization of CrfC-Venus with the mCherry-labeled clamp in single-focus MECS115-K cells. Upper panel: images of fluorescent foci and the morphology of a representative MECS115-K cell, which contained a single-clamp focus colocalized with CrfC focus at mid-cell, is shown. Lower panel: table displaying the percentages of cells containing colocalized foci.

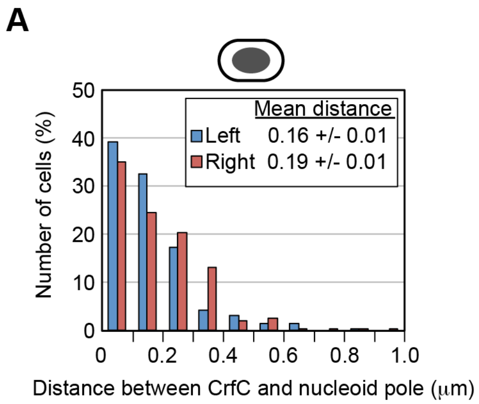

Supplementary Figure 3. Subcellular localization of CrfC and nucleoid. (A) Histogram of the distance between CrfC in the cell-polar area and the nucleoid pole in the cells analyzed for Figure 2C. The distance range (max, 1.0 μm) was divided into ten equal parts, and cells with distances corresponding to each range were scored. "Left" and "Right" indicate each pole of the nucleoid in Figure 2C. (B) Percentages of MECS133-K cells containing the indicated number of CrfC foci. Cells containing a single nucleoid (blue bar), a constricted nucleoid (magenta bar), or two nucleoids (green bar) were analyzed.

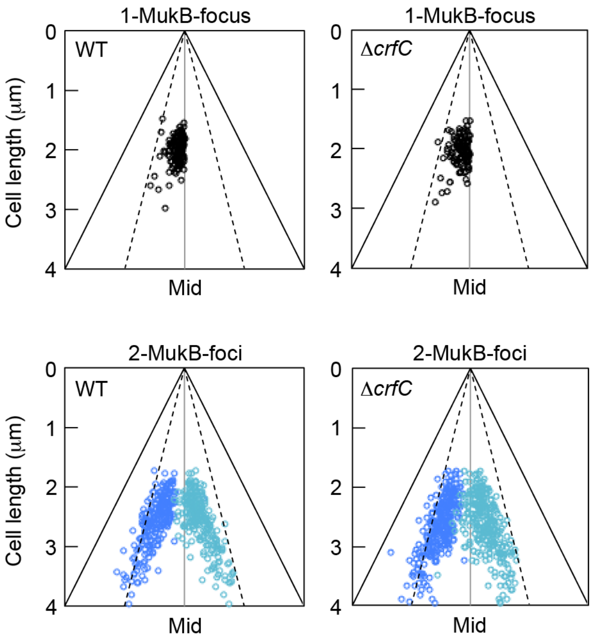

Supplementary Figure 4. Subcellular localization of MukB-mCherry foci. Subcellular positioning of MukB in MECS68 (wild-type *crfC*) and MECS83 (Δ*crfC*) cells. Cells bearing one (upper panel) or two MukB foci (lower panel) were analyzed. Dotted lines indicate quarter-cell positions, and black solid lines indicate the positions of the cell edges. Mid, mid-cell.

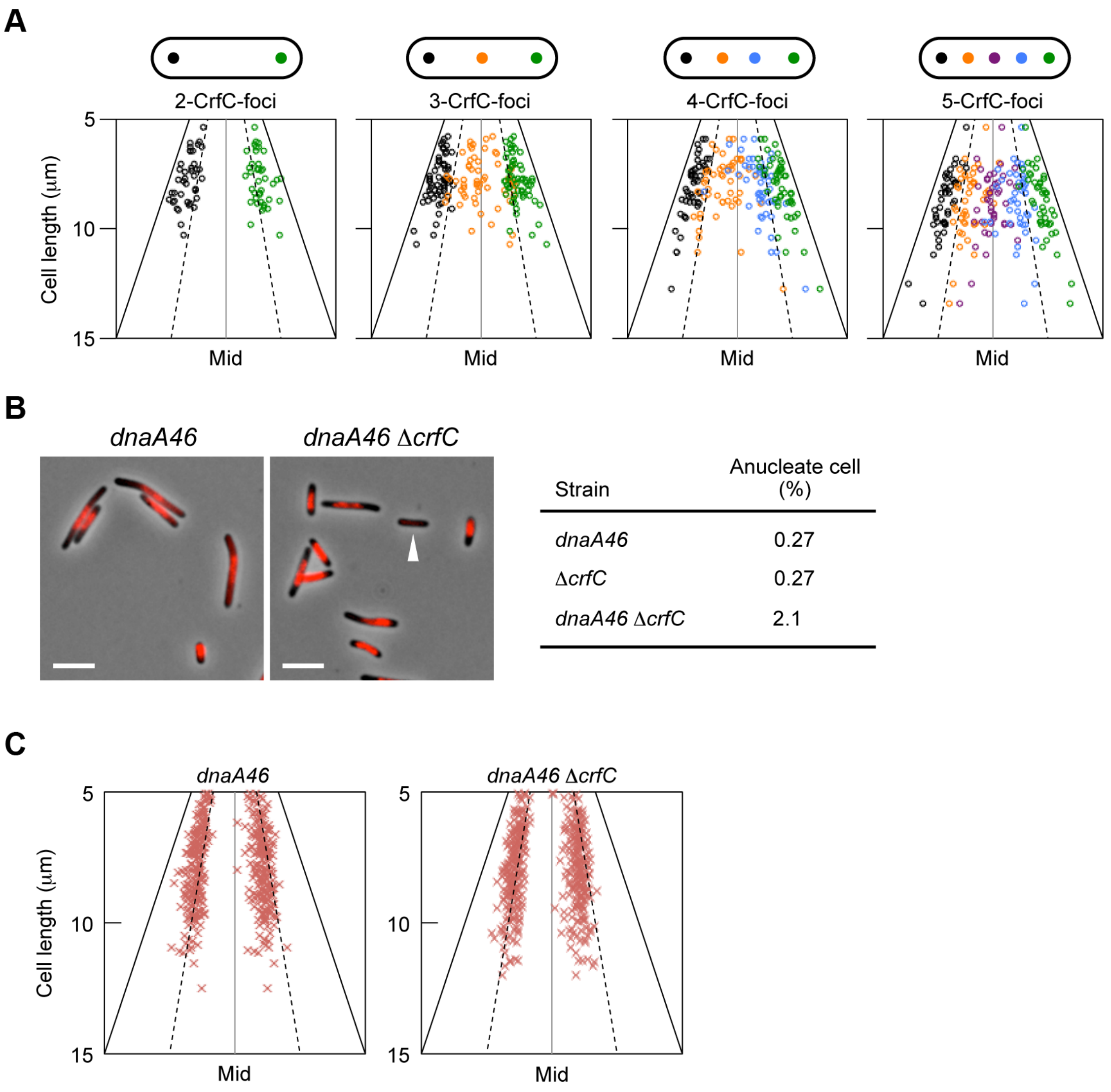

Supplementary Figure 5. Subcellular localization of CrfC and nucleoid in *dnaA46* filamentous cells. (A) Subcellular positioning of CrfC in MECS135 (*dnaA46*) filamentous cells bearing two to five CrfC foci. Dotted lines indicate quarter-cell positions, and black solid lines indicate the positions of cell poles. Mid, mid-cell. (B) Anucleate cell production of MECS135 (*dnaA46*) and MECS159 (Δ*crfC dnaA46*) mutants grown at 42°C for 2 hr. Left panel: Representative images of fluorescent foci and the morphology of MECS135 (*dnaA46*) and MECS159 (Δ*crfC dnaA46*) cells. White arrowhead indicates an anucleate cell. Scale bar is 5 μm. Right panel: Proportions of anucleate cells in MECS135 (*dnaA46*), MECS157 (Δ*crfC*) and MECS159 (Δ*crfC dnaA46*) mutants. In total, 366 (MECS135), 2217 (MECS157) or 373 (MECS159) cells were analyzed. (C) Subcellular positioning of the nucleoid poles in MECS135 (left panel) and MECS159 (right panel). Dotted lines indicate quarter-cell positions, and black solid lines indicate the positions of cell poles. Mid, mid-cell.

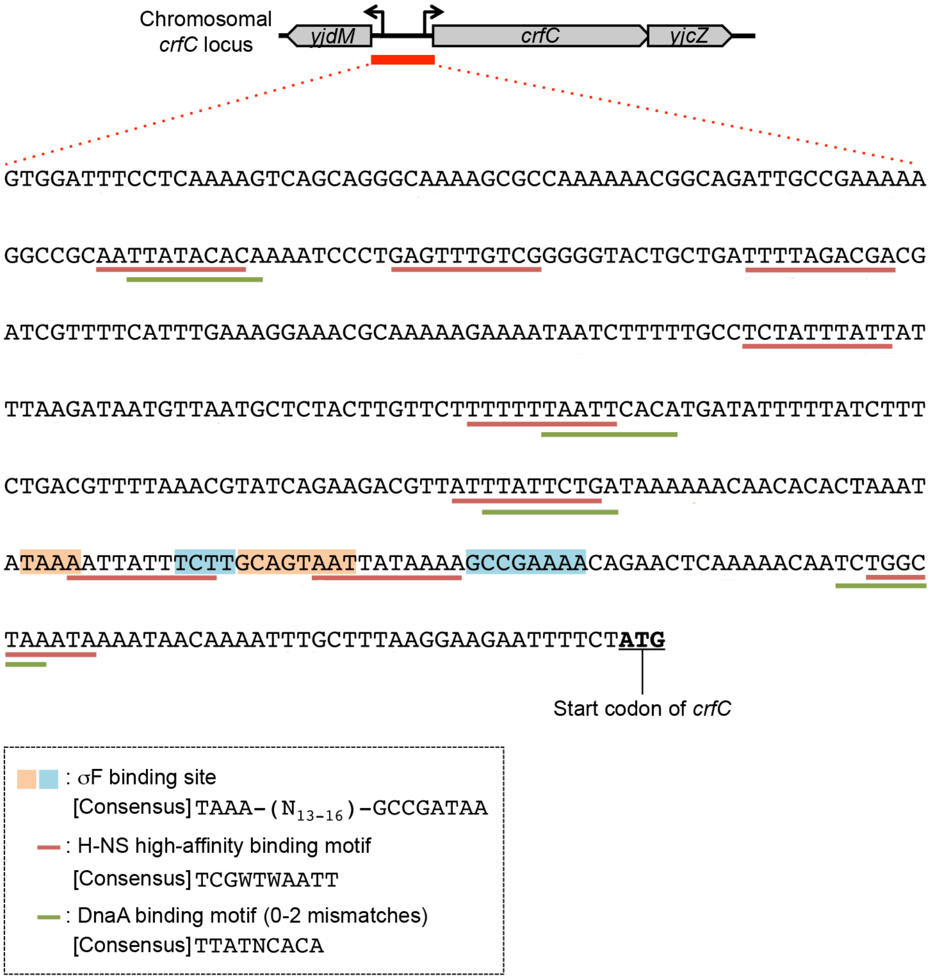

Supplementary Figure 6. Characteristics of the promoter region of the *crfC* gene. DNA sequence of the promoter region of the *crfC* gene. The sequence shows the 300 base pairs upstream of the *crfC* start codon (bold and underlined ATG). Orange and blue boxes indicate the consensus sequence for the σ^F (FliA)-binding site (Zhao et al., 2007). Magenta lines indicate the consensus sequence of the high-affinity binding site for H-NS (Lang et al., 2007). Green lines indicate the consensus sequence of DnaA box.

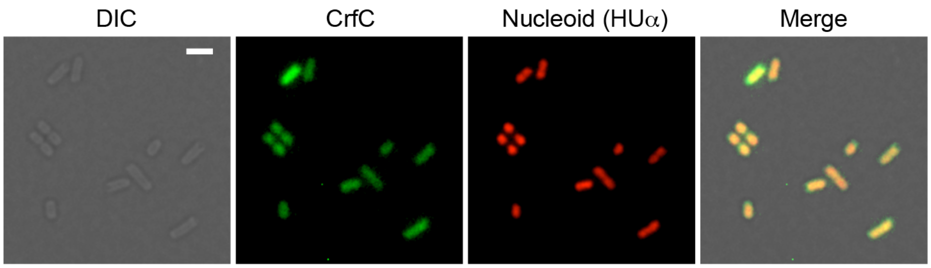

Supplementary Figure 7. Subcellular localization of CrfC overexpressed from pBR322 plasmid. Representative images of fluorescent foci and the morphology of MECS157-K (Δ*crfC hupA-cfp*) cells bearing pBR-*crfC-venus*. Scale bar is 2 μm.

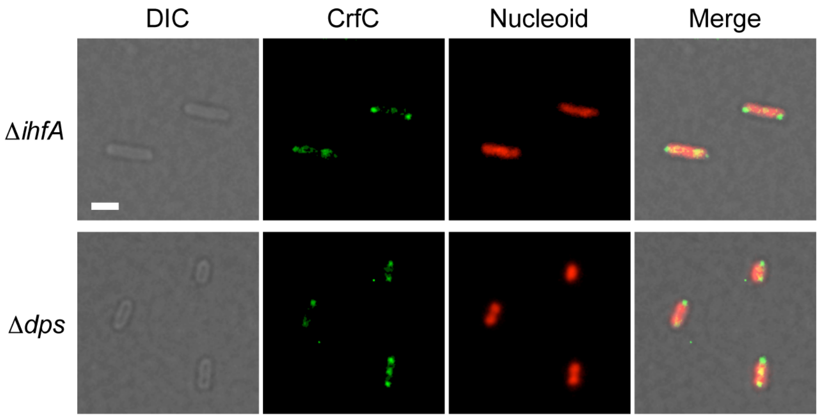

Supplementary Figure 8. Subcellular localization of CrfC in Δ*dps* or Δ*ihfA* cells. Representative images of fluorescent foci and the morphology of MECS173 (Δ*ihfA*) and MECS176 (Δ*dps*) cells are shown. Scale bar is 2 μm.

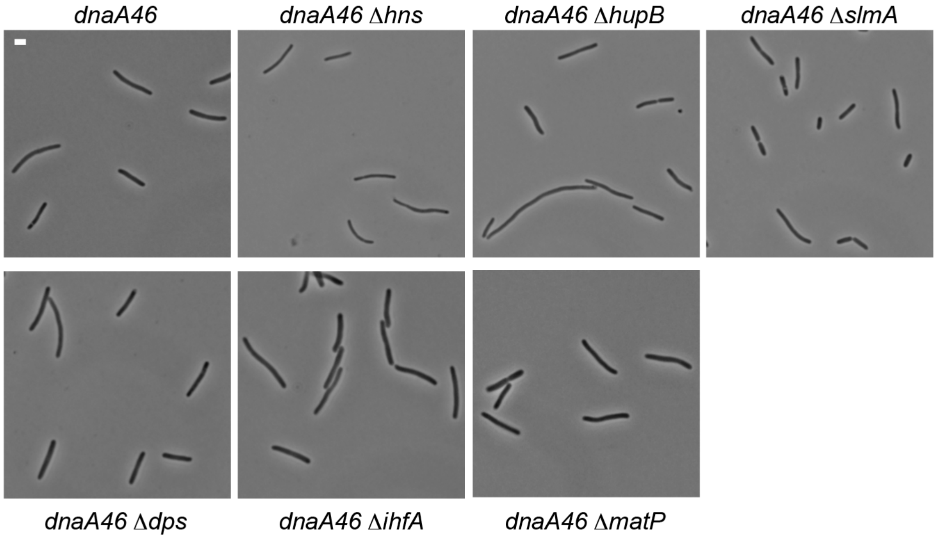

Supplementary Figure 9. Cell morphology of *dnaA46* strains. Representative morphological images of *dnaA46* strains [MECS135 (*dnaA46*), MECS174 (*dnaA46* ∆*hns*), MECS178 (*dnaA46* ∆*hupB*), MECS180 (*dnaA46* ∆*slmA*), MECS172 (*dnaA46* ∆*dps*), MECS175 (*dnaA46* ∆*ihfA*), and MECS160 (*dnaA46* ∆*matP*)] cells incubated at 42°C for 2 hr are shown. Scale bar is 2 μm.

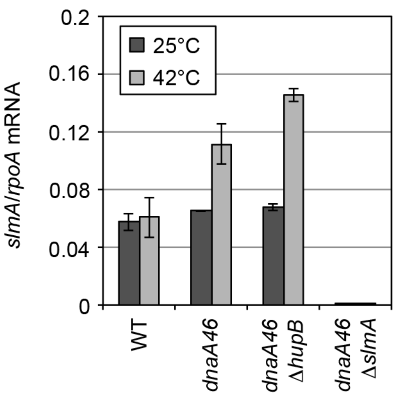

Supplementary Figure 10. mRNA level of *slmA* in *dnaA46* Δ*hupB* cells. Level of *slmA* mRNA in MECS135 (*dnaA46*) and MECS178 (*dnaA46* Δ*hupB*) cells. Cells were grown at 25°C (gray bars) and incubated at 42°C for an additional 2 hr (black bars). Error bars indicate SD (n=2).

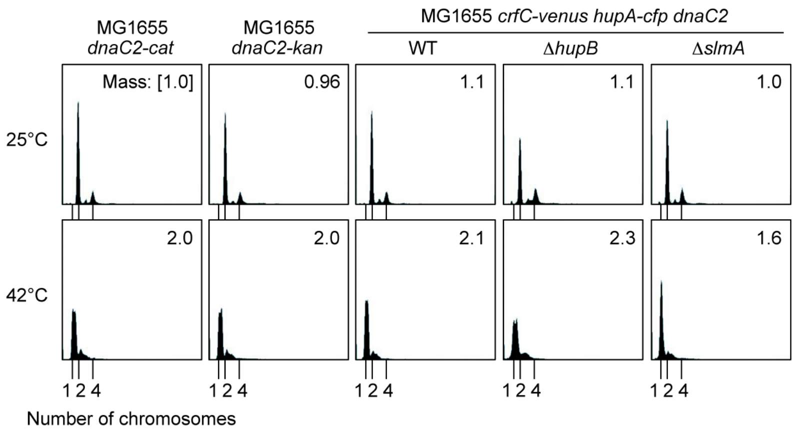

Supplementary Figure 11. Flow cytometry analysis of *dnaC2* mutants (KYA018, EYK37, MECS195, MECS196 and MECS197) incubated at 25 or 42°C in M9glu-caa medium. Cells were analyzed as described in Supplementary Figure 1.

Supplementary Table 1. List of *E. coli* strain

Strain	Genotype	Reference
MG1655	*ilvG rfb rph thyA rpsL* (wild-type)	Laboratory stock
MYU001-K	MG1655 Δ*crfC*	Ozaki et al., 2013
MYU002	MG1655 Δ*mukB*::*cat*	Ozaki et al., 2013
MYU008	MG1655 Δ*slmA*::*cat*	Ozaki et al., 2013
OZA004	MG1655 *frt-kan mCherry-dnaN*	Ozaki et al., 2013
OZA018	MG1655 Δ*crfC mCherry-dnaN*	This work
MIT125	MG1655 *dnaA46 tnaA::*Tn*10*	Noguchi et al., 2016
MIT147	MG1655 *seqA::Tn10*	Noguchi et al., 2016
AZ5159	*ftsZ84 leu-63*::Tn*10*	Kurokawa et al., 1999
JW0939	*rrnB* Δ*lacZ4787 hsdR514* Δ(*araBAD*)*567* Δ(*rhaBAD*)*568 rph-1* Δ*matP*::*frt-kan*	Keio collection, NIG
KMG5	MG1655 Δ*ihfA*::*frt-kan*	Kasho et al., 2014
KMG8	MG1655 Δ*hupB*::*frt-kan*	This work
KMG10	MG1655 Δ*dps*::*frt-kan*	This work
KX181	MG1655 Δ*hns*::*frt-kan*	This work
KYA018	MG1655 *dnaC2 zjj-18*::*cat*	Kasho and Katayama, 2013
EYK37	MG1655 *dnaC2 zjj-18*::*kan*	This work
MECS68	MG1655 *mukB-mCherry frt-kan*	This work
MECS83	MG1655 Δ*crfC mukB-mCherry frt-kan*	This work
MECS91-K	MG1655 *crfC-venus*	This work
MECS111-K	MG1655 *crfC-venus mukB-mCherry*	This work
MECS114	MG1655 *crfC-venus* Δ*mukB*::*cat*	This work
MECS115-K	MG1655 *crfC-venus mCherry-dnaN*	This work
MECS129	MG1655 *hupA-cfp frt-kan*	This work
MECS133-K	MG1655 *crfC-venus hupA-cfp*	This work
MECS135	MG1655 *crfC-venus hupA-cfp dnaA46 tnaA*::Tn*10*	This work
MECS145	MG1655 *crfC-venus* Δ*matP*::*frt-kan*	This work
MECS150	MG1655 *crfC-venus hupA-cfp ftsZ84 leu-63*::Tn*10*	This work
MECS157	MG1655 Δ*crfC hupA-cfp*	This work
MECS159	MG1655 Δ*crfC hupA-cfp dnaA46 tnaA*::Tn*10*	This work
MECS160	MG1655 *crfC-venus hupA-cfp* Δ*matP*::*frt-kan dnaA46 tnaA*::Tn*10*	This work
MECS171	MG1655 *crfC-venus hupA-cfp* Δ*hns*::*frt-kan*	This work
MECS172	MG1655 *crfC-venus hupA-cfp* Δ*dps*::*frt-kan dnaA46 tnaA*::Tn*10*	This work
MECS173	MG1655 *crfC-venus hupA-cfp* Δ*ihfA*::*frt-kan*	This work
MECS174	MG1655 *crfC-venus hupA-cfp* Δ*hns*::*frt-kan dnaA46 tnaA*::Tn*10*	This work
MECS175	MG1655 *crfC-venus hupA-cfp* Δ*ihfA*::*frt-kan dnaA46 tnaA*::Tn*10*	This work
MECS176	MG1655 *crfC-venus hupA-cfp* Δ*dps*::*frt-kan*	This work

(Supplementaly Table 1. continued)

Strain	Genotype	Reference
MECS177	MG1655 *crfC-venus hupA-cfp ΔslmA*::*frt-kan*	This work
MECS178	MG1655 *crfC-venus hupA-cfp ΔslmA*::*frt-kan dnaA46 tnaA*::Tn*10*	This work
MECS179	MG1655 *crfC-venus hupA-cfp ΔhupB*::*frt-kan*	This work
MECS179-K	MG1655 *crfC-venus hupA-cfp ΔhupB*	This work
MECS180	MG1655 *crfC-venus hupA-cfp ΔhupB*::*frt-kan dnaA46 tnaA*::Tn*10*	This work
MECS193	MG1655 *crfC-venus hupA-cfp seqA*::Tn*10*	This work
MECS195	MG1655 *crfC-venus hupA-cfp dnaC2 zjj-18*::*cat*	This work
MECS196	MG1655 *crfC-venus hupA-cfp ΔhupB dnaC2 zjj-18*::*cat*	This work
MECS197	MG1655 *crfC-venus hupA-cfp ΔslmA*::*cat dnaC2 zjj-18*::*kan*	This work

Supplementary Table 2. List of plasmids

Plasmid	Description	Reference
pTH59	*cfp-frt-kan*	Hatano and Niki, 2010
pTH1017	*venus-frt-kan*	Gift from H. Niki
pTH1161	*mCherry-frt-kan*	Ozaki et al., 2013
pBR-*crfC-venus*	pBR322-*crfC-venus*	This work

Supplementary Table 3. Sequences of PCR primers

Name	Sequence (5' to 3')
SP123	AACGCTTCCAGGAACTGACGAAGCGCCTTCTCAGGCGAGTATGGTGAGCAAGGGCGAGGAG
SP124	TTTCGGAAAAAGAAAAGGCGGCATTGCTGCCGCCTTAATTGTGTAGGCTGGAGCTGCTTC
SP140	CGATGATATTCAAACACTTTTCACGGCAGAACGATATGTGAGCAAGGGCGAGGAGCT
SP186	AAACCACCCCTTCGTTAAAACTGTTCACTGCCACGCAATCGTGTAGGCTGGAGCTGCTTC
SP190	ATTTGTTTCTGGCAAGGCACTGAAAGACGCAGTTAAGGTGAGCAAGGGCGAGGAGCT
GFP-b	GACTCCAGCACGCGACCGGGGCCGTCAAGTAACGTCTTGGGTGTAGGCTGGAGCTGCTTC
RT-rpoA-L	CCCAGAGTATGGCCAAAGCC
RT-rpoA-U	CTGTGACAGAGTTTCTAAAACCGC
SP234	TGGCTTTACCCTGGAGTCAG
SP235	TGGTTGATTTCCCTGCTTTC
SP256	CAAAACTGGCCGCCTCTGTC
SP257	CTTTCAGAATCAGGTTGATGCGAG
